# Diagnosis of malignant body fluids via cancer-universal methylation in cell-free DNA

**DOI:** 10.1172/jci.insight.175482

**Published:** 2024-04-08

**Authors:** Zhanrui Mao, Shihua Dong, Yu Yan, Chengyang Wang, Wei Li, Lu Wang, Chengchen Qian, Yuanlin Song, Lin Tong, Wenqiang Yu

**Affiliations:** 1 Institutes of Biomedical Sciences, Shanghai Public Health Clinical Center, Cancer Metastasis Institute, and Department of General Surgery, Huashan Hospital, Shanghai Medical College, Fudan University, Shanghai, China.; 2 Shanghai Epiprobe Biotechnology Co., Ltd, Shanghai, China.; 3 Department of Pulmonary and Critical Care Medicine, Zhongshan Hospital, Fudan University, Shanghai, China.; 4 Shanghai Respiratory Research Institute, Shanghai, China.

**Keywords:** Genetics, Oncology, Cancer, Epigenetics, Molecular diagnosis

## Abstract

**BACKGROUND:**

Differentiating malignant from nonmalignant body fluids remains a clinical challenge because of the unsatisfying performance of conventional cytology. We aimed to improve the sensitivity and ubiquity of cancer cell detection by assaying universal cancer–only methylation (UCOM) markers in supernatant cell-free DNA (cfDNA).

**METHODS:**

An observational prospective cohort including 1,321 nonmalignant and malignant body fluids of multiple cancers was used to develop and validate a cfDNA UCOM methylation diagnostic assay. All samples were divided into 2 portions for cytology and supernatant cfDNA methylation analysis.

**RESULTS:**

The significant hypermethylation of a potentially novel UCOM marker, TAGMe, together with the formerly reported *PCDHGB7*, was identified in the cfDNA of malignant body fluid samples. The combined model, cell-free cancer-universal methylation (CUE), was developed and validated in a prospective multicancer cohort with markedly elevated sensitivity and specificity, and was further verified in a set containing additional types of malignant body fluids and metastases. In addition, it remained hypersensitive in detecting cancer cells in cytologically negative malignant samples.

**CONCLUSION:**

cfDNA methylation markers are robust in detecting tumor cells and are applicable to diverse body fluids and tumor types, providing a feasible complement to current cytology-based diagnostic analyses.

**TRIAL REGISTRATION:**

This study was registered at Chictr.org.cn (ChiCTR2200060532).

**FUNDING:**

National Natural Science Foundation of China (32270645, 31872814, 32000505, 82170088), the National Key R&D Program of Ningxia Hui Autonomous region (2022BEG01003), Shanghai Municipal Key Clinical Specialty (shslczdzk02201), Science and Technology Commission of Shanghai Municipality (20DZ2261200, 20DZ2254400), and Major Special Projects of Basic Research of Shanghai Science and Technology Commission (18JC1411101).

## Abstract 2

Cancer-universal methylation in cell-free DNA is highly sensitive and consistent across cancer types in detecting malignant body fluids in a prospective multi-cancer cohort.

## Introduction

Metastases of the pleura, peritoneum, and others occur frequently in patients with malignant cancers, in which the dissemination of tumor materials, including cells, cell-free nucleic acids, etc., systemically leverage several serous membrane fluids and circulate to distant organs (1, 2). Thus, detecting tumor cells in specific body fluids could help determine the presence of cancer metastases in corresponding sites (3–5), and enable prompt diagnosis and early treatment to optimize life quality and survival for patients with advanced cancer (6).

However, the diagnosis of malignant body fluids (MBFs) remains a substantial challenge, as the current gold standard, cytological analysis, is rather unsatisfying given its lack of sensitivity (4, 7). For example, cytological analysis has demonstrated rather limited overall sensitivity for malignant pleural effusions (MPEs), ranging from 40% to 87%, owing to variations in study design, methodology, and cytopathologists (7–9). Additionally, the estimation of sensitivity is variable among different types of cancer, which further compromises its clinical practicability (10). Other routine methods like radiological examination and tumor markers (i.e., CEA, NSE, CA-125) also have respective drawbacks and thus, fail to establish a definitive diagnosis (11–14). For instance, false-positive NSE results are common in the clinic due to specimens left for more than an hour before testing. As a result, more invasive biopsies such as thoracoscopy or CT-guided percutaneous biopsy are commonly required to perform differential diagnosis, which inevitably delays prompt intervention for these patients (7). Therefore, assays with high sensitivity and specificity for the identification of MBF are of great clinical importance.

DNA methylation is a vital epigenetic regulator of gene expression and tissue differentiation during tumorigenesis, and has been intensively explored in diverse cancers in recent decades (15–18). Methylation aberration in circulating tumor cell-free DNA (cfDNA) is one of the most extensively studied cancer markers in liquid biopsy samples (19–21). Its application as a biomarker for cancer screening has been evaluated, yet the application is still limited to specific tumor types (e.g., colorectal cancer) (22, 23). Nevertheless, considering the diversity of tumor primary sites, subtypes, and sample properties present in clinical diagnosis, the much-needed assays for MBF detection are also required to capture shared patterns of malignancy. Previously, we have proposed the concept of universal cancer–only methylation (UCOM), a collection of cancer-specific epigenetic features shared among most cancer types (24, 25). The clinical value of several markers has already been demonstrated in early diagnosis of specific cancer types, including lung cancer, cervical cancer, and endometrial cancer (24, 26, 27). However, the clinical utility of their pan-cancer characteristics remains to be demonstrated in a real-world study with diverse tumor types.

In this study, we first investigated the feasibility of cfDNA UCOM markers, including what we believe is a novel identified marker, TAGMe, and formerly reported *PCDHGB7*, to distinguish MPEs from nonmalignant ones (*n* = 62). Next, we constructed and tested a diagnostic model, cfDNA cancer-universal methylation (CUE), in a blinded, prospective clinical study, including 1,158 pleural effusion (PE) and ascites samples of patients with pleural and peritoneal metastases of more than 20 types of tumors. Subsequently, its efficacy was further validated in other types of body fluids (*n* = 163). The clinical performance of the CUE model was also verified in malignant samples with negative cytology (*n* = 38).

## Results

**Figure 1:**
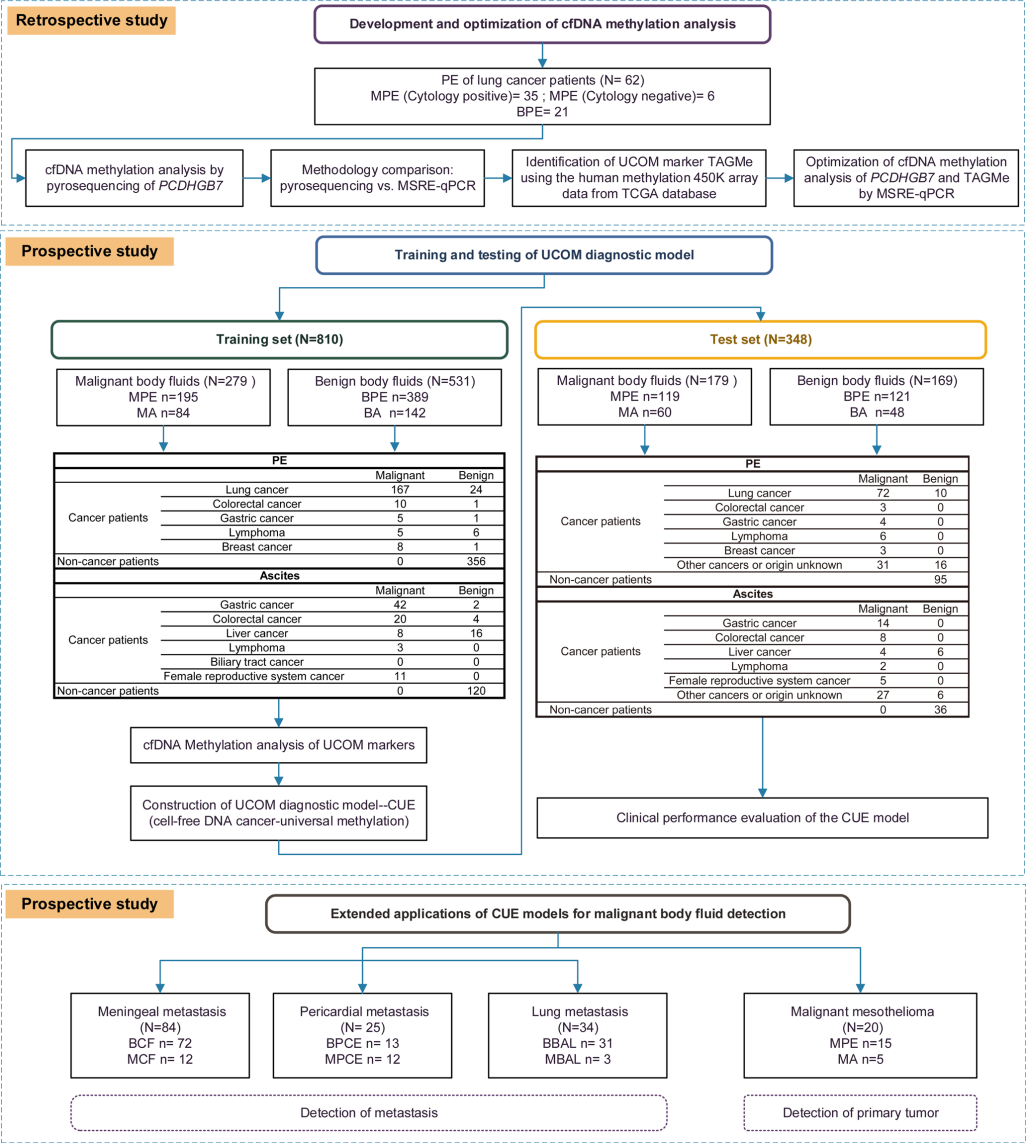
Diagram of workflow. PE, pleural effusion; MPE, malignant pleural effusion; BFE, benign pleural effusion; MA, malignant ascites; BA, benign ascites; BCF, benign cerebrospinal fluid; MCF, malignant cerebrospinal fluid; BPCE, benign pericardial effusion; MPCE, malignant pericardial effusion; BBAL, benign bronchoalveolar lavage fluid; MBAL, malignant bronchoalveolar lavage fluid.

A diagrammatic workflow of the study design and patient enrollment are shown in Figure 1 and Supplemental Figure 1 (supplemental material available online with this article; https://doi.org/10.1172/jci.insight.175482DS1).

### Aberrant cfDNA methylation in PEs associated with malignancy.

**Figure 2:**
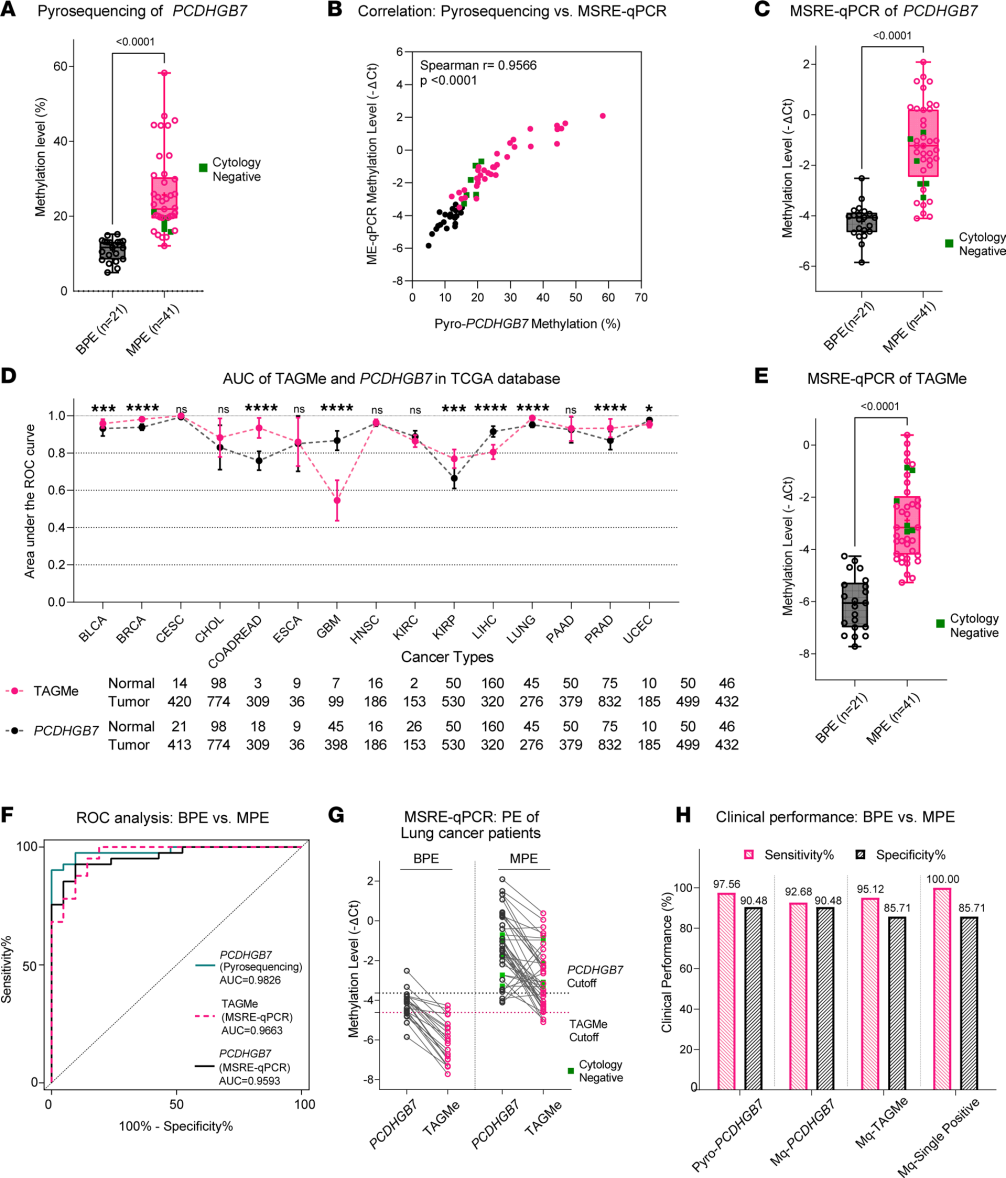
UCOM markers demonstrated to identify tumor cells using cfDNA in malignant pleural effusion (MPE). (**A**) Significant cfDNA hypermethylation of *PCDHGB7* was detected in MPE samples from lung cancer patients by pyrosequencing. (**B**) MSRE-qPCR–based results highly correlated with the methylation percentages using cfDNA pyrosequencing. Spearman’s *r* correlation and *P* value were calculated using GraphPad Prism 9.3.0. (**C**) MSRE-qPCR simultaneously revealed *PCDHGB7* cfDNA hypermethylation in MPE samples. (**D**) ROC analysis demonstrates complementary performance of the marker identified in this study, TAGMe, and *PCDHGB7* across multiple cancer types in TCGA database. *P* values were calculated by pair-wise comparison of ROC curves test with SPSS 20.0. *P < 0.05; ***P < 0.001; ****P < 0.0001.NS, not significant. (**E**) cfDNA hypermethylation of TAGMe was also validated in MPE samples by MSRE-qPCR. (**F**) ROC analysis demonstrated the performance of the 3 assays. (**G**) Complementary results for 2 markers were found in the same sample. (**H**) The sensitivity can be maximized by a 2-marker detection in a single-positive-for-positive method. Black circles: nonmalignant samples; pink circles: malignant samples with positive cytology; green squares: malignant samples in which cytology failed to detect tumor cells. BLCA, bladder cancer; BRCA, breast cancer; CESC, cervical cancer; CHOL, bile duct cancer; COADREAD, colon and rectal cancer; ESCA, esophageal cancer; GBM, glioblastoma; HNSC, head and neck cancer; KIRC, kidney clear cell carcinoma; KIRP, kidney papillary cell carcinoma; LIHC, liver cancer; LUNG, lung cancer; PAAD, pancreatic cancer; PRAD, prostate cancer; UCEC, endometrioid cancer. *P* values in **A**, **C**, and **E** were calculated using a 2-tailed, nonparametric Mann-Whitney test as determined by GraphPad Prism 9.3.0.

The aberrant methylation status of several UCOM markers has been demonstrated to reflect tumors and precancerous lesions in exfoliated cells. But its utility in indicating the presence of tumor-origin cell-free nucleic acids has yet to be established. As shown in Figure 1, in this study, we first verified the cfDNA methylation status of a formerly reported marker, *PCDHGB7*, in 62 MPEs and benign PEs (BPEs), using bisulfite-PCR pyrosequencing as the gold standard for methylation status (Supplemental Table 1). As expected, distinct cfDNA hypermethylation was found in the MPE group (*n* = 41), even for 6 of the malignant samples that were once miscategorized as negative by cytology analysis (Figure 2A). However, pyrosequencing is time consuming and expensive; thus, to achieve high stability, rapidity, and cost effectiveness of clinical assays, we further investigated whether the quantitative detection of cfDNA methylation could be conducted using the methylation-sensitive restriction enzyme combined with real-time fluorescent quantitative PCR (MSRE-qPCR) method. We optimized the previously established experimental system to prevent variability in enzyme cleavage efficiencies from impairing the reproducibility upon assaying trace amounts of small fragmented DNA samples. As shown in Figure 2B, comparative tests revealed a significant correlation between the ΔCt yielded by MSRE-qPCR and the methylation percentage of pyrosequencing (*r* = 0.9566, 95%CI: 0.9278–0.9741, *P* < 0.0001; lower ΔCt suggests higher methylation percentage). However, it was also noted that, while the difference in *PCDHGB7* methylation detected by MSRE-qPCR remained significant between the 2 groups (*P* < 0.001), it was not as distinguishable as pyrosequencing for cytologically negative MPEs (Figure 2C).

To further improve the efficiency of cfDNA methylation detection in PEs, we identified a UCOM marker, TAGMe, a significantly differentially methylated region (DMR) located in the intergenic region in chromosome 3p26.1, that outperformed *PCDHGB7* in several cancer types, including lung, colorectal, kidney, prostate, and bile duct cancers (Figure 2D and Supplemental Tables 2 and 3). Further verification by MSRE-qPCR in cancer cell lines and solid tissues across 15 types of cancers confirmed that TAGMe hypermethylation is a pan-cancer marker (Supplemental Figure 2). Moreover, the MSRE-qPCR results of TAGMe also exhibited enhanced detection capability of cytology-negative MPEs (Figure 2E). ROC analysis also showed that the MSRE-qPCR analysis for TAGMe was superior to that for *PCDHGB7*, with similar performance to the pyrosequencing (Figure 2F). Detecting the 2 UCOM markers simultaneously in the same sample can further increase the sensitivity, while slightly compromising the specificity in a simple single-positive-as-positive combination, which is more advantageous in addressing the lack of sensitivity of cytology (Figure 2, G and H). Altogether, these results suggest that cfDNA hypermethylation of TAGMe and *PCDHGB7* are cancer-specific features in MPE samples from lung cancer patients and hold potential as universal markers for malignancy in body fluids.

### Development of the cfDNA UCOM diagnostic model in a prospective multicancer clinical cohort.

To further assess the cancer-specific cfDNA methylation in various types of cancer and body fluids, the methylation status of TAGMe and *PCDHGB7* was analyzed in a prospective multicancer clinical cohort involving 1,158 samples (Figure 1 and Supplemental Figure 1). The cohort consisted of 314 (27.12%) MPEs, 144 (12.44%) malignant ascites (MA), 510 (44.41%) BPEs, and 190 (16.41%) benign ascites (BA) with definitive diagnosis. These samples represent 551 patients with lung cancer (23.92%), gastric cancer (5.78%), colorectal cancer (4.23%), liver cancer (3.45%), lymphoma (1.72%), breast cancer (1.03%), gynecological cancer (1.72%), and other cancer (5.69%), as well as 607 patients with benign diseases (52.41%). The samples were assigned to the training and test sets in a ratio of 7:3, and the demographic and clinical characteristics of the 2 sets are summarized in Tables 1 and 2.

**Figure 3:**
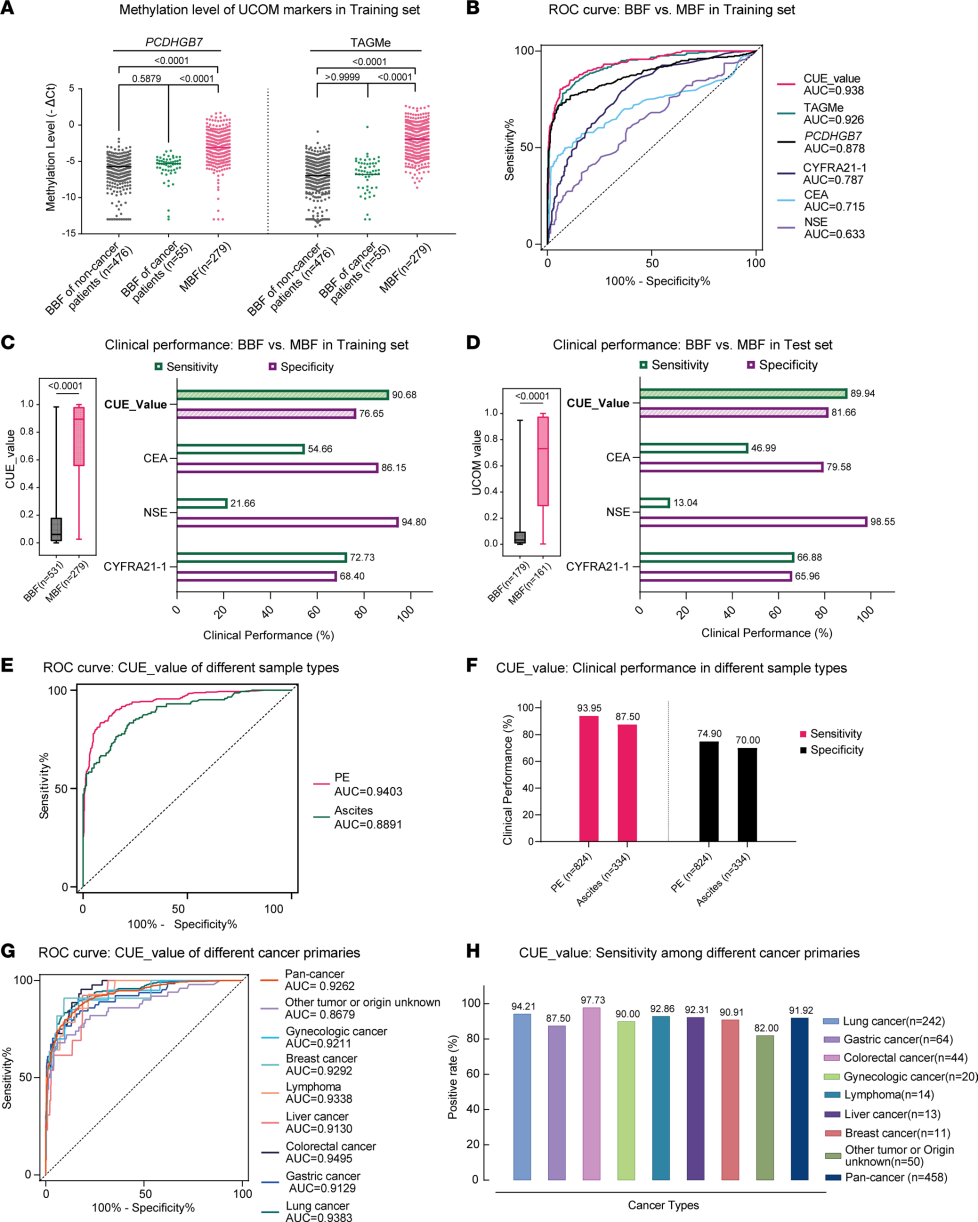
Development and validation cfDNA UCOM diagnostic model in a prospective multicancer cohort. (**A**) Methylation levels of cfDNA UCOM markers are significantly higher in MPE and ascites than in benign ones from patients with benign diseases or cancers in the training set. BBF, benign body fluid. (**B**) ROC analysis shows that the CUE model yielded the highest AUC compared with a single UCOM marker as well as the conventional tumor markers. (**C**) The clinical performance of the CUE model using an optimal threshold is illustrated. (**D**) Consistent performance was observed in the test set at the same threshold. (**E** and **G**) The AUC of the CUE model in different types of sample (**E**) and primary cancer (**G**) in training and test sets. (**F** and **H**) The clinical performance of the CUE model between sample types (**F**) and cancer types (**H**) was evaluated. *P* values in **A**, **C**, and **D** were calculated by the 2-tailed, nonparametric Mann-Whitney test, whereas those in **B** were determined by the Kruskal-Wallis test, and calculations were all performed via GraphPad Prism 9.3.0.

In the training set, *PCDHGB7* and TAGMe were both significantly hypermethylated in the MBF group (Figure 3A). These 2 UCOM markers also proved to be independent diagnostic factors in a multivariate analysis including clinical variables (TAGMe: hazard ratio [HR] = 2.366, 95%CI = 1.793–3.122, *P* < 0.0001; *PCDHGB7*: HR = 1.458, 95%CI = 1.131–1.879, *P* = 0.004; Supplemental Figure 3A). Meanwhile, hypermethylation was not observed in nonmalignant samples from cancer patients without corresponding metastatic or in situ tumors, thus highlighting their specificity for detecting localized metastasis (Figure 3A). Specifically, the cancer universality of *PCDHGB7* and TAGMe hypermethylation was demonstrated by the statistically significant differences generally found in malignant and nonmalignant samples from patients with different cancer types (Supplemental Figure 3, B and C). Furthermore, to develop an enhanced cfDNA UCOM assay for detection of malignancy, binary logistic regression analysis was performed to build a diagnostic model, CUE, consisting of these 2 UCOM markers (see Methods). The CUE model yielded superior performance compared with other conventional markers like CEA, NSE, and CYFRA21-1 or any single UCOM marker (AUC = 0.9364, 95%CI = 0.9189–0.9540; Figure 3B). The sensitivity and specificity of the CUE model was 93.19% (95%CI = 89.61%–95.6%) and 73.07% (95%CI = 69.14%–76.67%), indicating its potential as a diagnostic assay for pleural and peritoneal metastases from multiple cancer types (Figure 3C and Table 3).

### Validation of the clinical performance and cancer universality of the CUE diagnostic model.

To evaluate the clinical performance of the CUE diagnostic model, the test set (*n* = 348) included PE samples and ascites from patients with more than 20 types of cancer (Tables 1 and 2), which were analyzed by MSRE-qPCR. The results showed similar hypermethylation of both UCOM markers in malignant samples (Supplemental Figure 3D). The CUE model remains the best classifier in comparison with routine cancer markers (AUC = 0.9275, 95%CI = 0.9005–0.9546; Supplemental Figure 3E). When adapting the same threshold as in the training set, the CUE model yielded similar sensitivity of 89.94% (95%CI = 84.67%–93.54%) and specificity of 81.66% (95%CI = 75.14%–86.77%), which demonstrated the stability of its clinical performance (Figure 3D and Table 3).

Subsequently, aiming to complement the insufficient sensitivity of current assays in certain circumstances, we aimed to investigate whether the performance of the CUE model varies across sample and cancer types. The subgroup statistical analysis was applied to the combined data set of training and test sets. The CUE model performed well in both PE (AUC = 0.9403) and ascites (AUC = 0.8891) samples, although the diagnostic performance was higher in PEs (Figure 3, E and F, and Supplemental Figure 3F). Additionally, high levels of CUE values were also frequently observed in subgroups stratified by tumor types (Supplemental Figure 3G), with the AUC varying between 0.8679 (other cancers or origin unknown) and 0.9495 (colorectal cancer) (Figure 3G and Supplemental Figure 3G). And the positive rates of the CUE model ranged from 82.00% (other cancers or origin unknown) to 97.73% (colorectal cancer) at the same threshold as before (Figure 3H). Overall, these findings support the robust performance of the CUE model in determining pleural and peritoneal metastases from various cancer types, which is applicable to different body fluid types.

### Extended applications of the CUE model for MBF detection.

**Figure 4:**
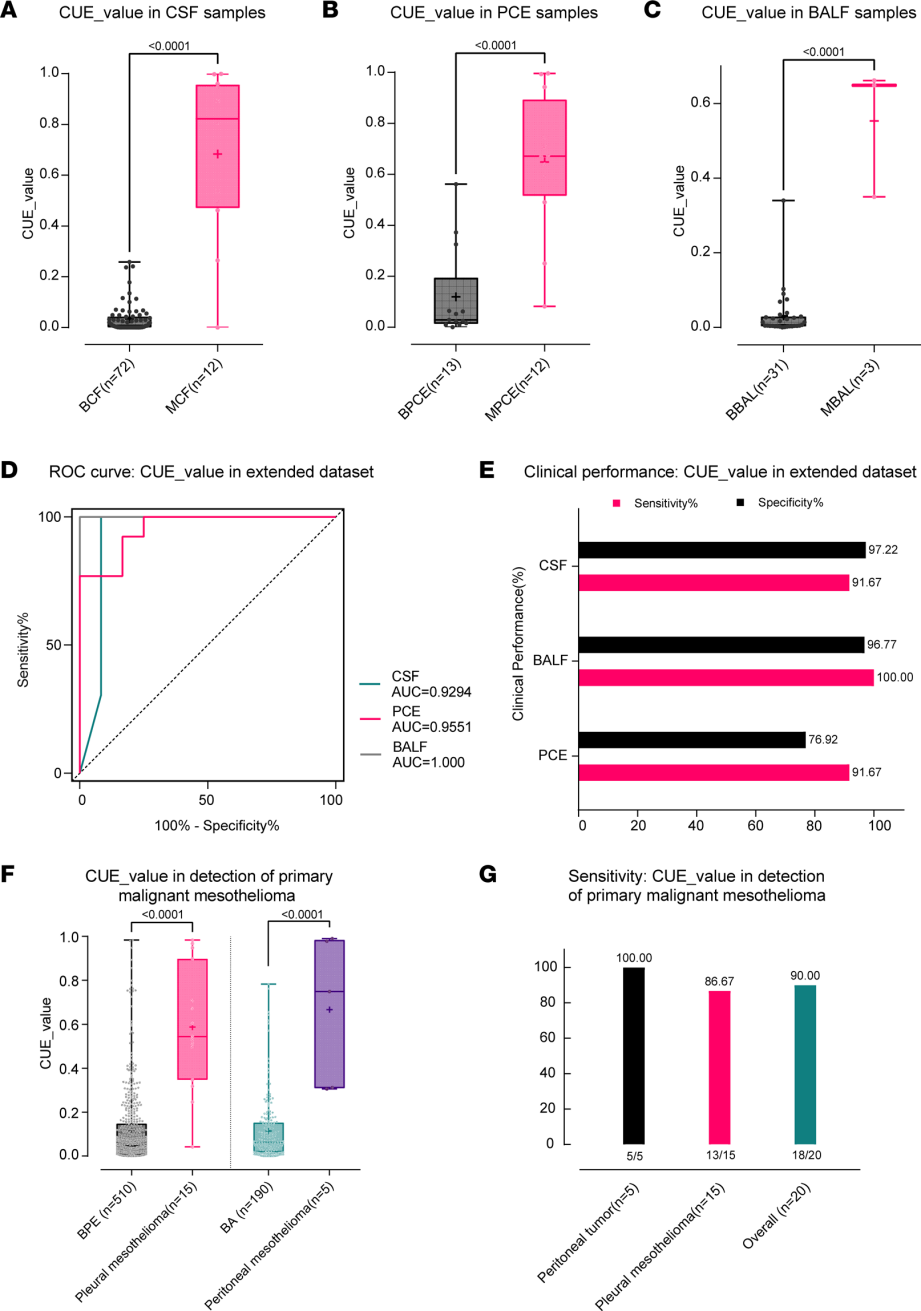
Additional evidence of the CUE model’s capacity for malignancy detection. (**A**–**C**) cfDNA methylation measured by CUE value was significantly associated with meningeal, pericardial, and pulmonary metastases in CSF (**A**), PCE (**B**), and BALF (**C**) samples, respectively. (**D**) The AUC of the CUE model for detection of different metastases is shown, as well as the clinical performance (**E**). The CUE model also serves to identify malignant mesothelioma in effusion samples (**F**) with robust sensitivity (**G**). *P* values in **A**–**C** and **F** were calculated using a 2-tailed, nonparametric Mann-Whitney test as determined by GraphPad Prism 9.3.0.

Following the validation of CUE model to detect the pleuroperitoneal metastases in PE and ascites originating from various cancer types, we further explored the application of the CUE model to additional types of body fluids and metastases (Figure 1 and Supplemental Figure 1). The extended data set consisted of 84 cases of cerebrospinal fluid (CSF), 25 cases of pericardial effusion (PCE), 34 cases of bronchoalveolar lavage fluid (BALF), and 20 cases of PE and ascites from patients with malignant mesothelioma (Supplemental Table 4). cfDNA hypermethylation was observed in CSF samples from patients with meningeal metastases, as indicated by the UCOM marker as well as the CUE value derived from the CUE model (Figure 4A and Supplemental Figure 4A). Similar results were also found in PCEs, demonstrating its diagnostic value for pericardial metastases (Figure 4B and Supplemental Figure 4B). Moreover, its feasibility in detecting pulmonary metastases in BALF samples was also illustrated (Figure 4C and Supplemental Figure 4C). Collectively, the CUE model exhibits capacity to detect metastatic tumor cells in 3 sample types: CSF, PCE, and BALF (AUC = 0.9294, 0.9551, and 1.000, respectively). Using the same threshold, we observed sensitivities exceeding 90% in all, and specificities were relatively low in PCE samples (76.92%), but higher than 95% in others (Figure 4, D and E).

Malignant mesothelioma is a rare malignant tumor that is frequently accompanied by PEs and abdominal effusions and, due to the low sensitivity of cytology, most patients require tissue biopsy to make a definitive diagnosis (7, 28). Therefore, we intended to improve the diagnosis of malignant mesothelioma through the CUE model. We found that cfDNA methylation was significantly higher in PE (*n* = 15) and ascites (*n* = 5) samples from malignant pleural and peritoneal mesothelioma patients than in benign body fluid samples from the training and test sets (Figure 4F and Supplemental Figure 4D). Of these 20 patients, 6 were found to be negative by cytological testing (30%), abnormal cells were found in 5 cases (25%), and tumor cells were found in 9 cases (45%), while the overall sensitivity of the CUE model was 90.00% (16 out of 18) (Figure 4G). In particular, the sensitivity of detecting peritoneal mesothelioma in ascites was 100% (5 out of 5), while the sensitivity of diagnosing pleural mesothelioma in PEs was 86.67% (13 out of 15). Overall, the UCOM markers and model proved to be generally applicable in various subgroups involved in this study. In summary, the feasibility of detecting tumor cells in body fluids by the CUE model has been further verified in more types of cancer metastasis and in situ mesothelioma diagnosis.

### UCOM model acts as a complementing test for cytology to improve diagnostic sensitivity.

**Figure 5:**
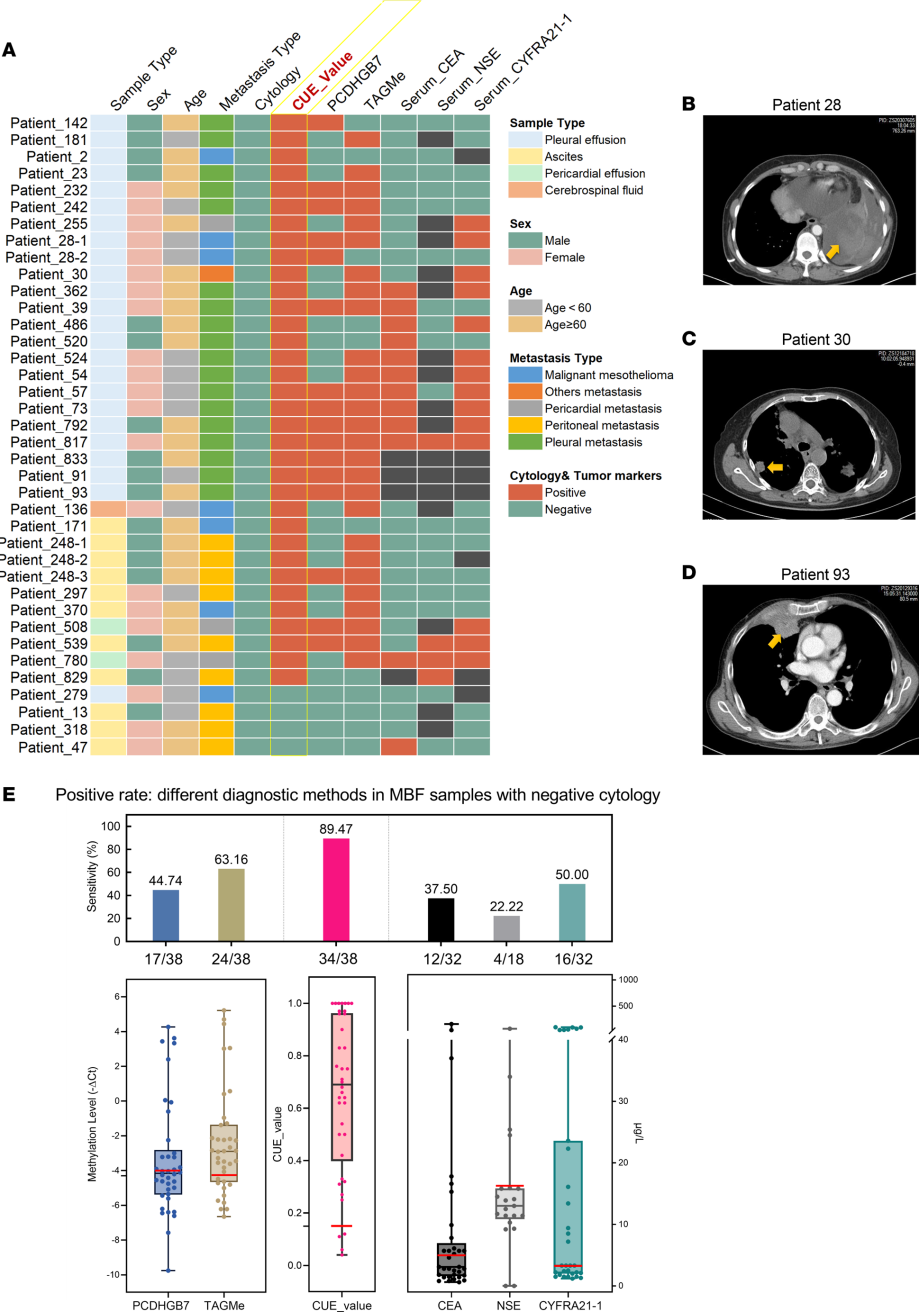
Performance of the CUE model in malignant samples with cytologically undetectable cancer cells. (**A**) The clinical information of 38 malignant body fluids that were diagnosed as negative by cytology analysis. (**B**–**D**) The CT images of 3 typical cases are demonstrated. (**B**) Primary malignant pleural mesothelioma. (**C**) Recurrent lung adenocarcinoma. (**D**) Pleural metastasis of laryngeal cancer. Yellow arrow: tumor site. (**E**) The positive rate of the CUE model was optimal compared with an individual UCOM marker and conventional cancer markers.

Positive cytological analysis is viewed as the gold standard for malignancies in body fluids, although the negative results can not exclude malignancies. To demonstrate that the CUE model could capture the trace of tumor cells left out by cytology, a total of 38 body fluids that were identified as cytologically negative from patients with a final diagnosis of metastasis or a primary carcinoma in situ were pooled for further analysis (Supplemental Table 5). The pathological information and test results are shown in Figure 5A, which included 24 cases of MPE, 11 cases of MA, 2 cases of malignant pericardial effusion (MPCE), and 1 case of malignant CSF (MCF). Most cytologically negative MPE samples were from patients with pleural metastases from lung cancer (9 cases), in addition to 4 cases originating from malignant pleural mesothelioma, 3 lymphoma metastases, as well as metastases from breast, bone, and colon cancers. And the MA samples contained 6 cases of metastasis from hepatocellular carcinoma, 2 cases of malignant peritoneal mesothelioma, 2 cases of sarcoma, and 1 case of colon-origin metastasis, respectively. In addition, MPCE and MCF samples represented pericardial and meningeal metastases, respectively from lung adenocarcinoma.

The CT images of 3 typical cases are demonstrated in Figure 5, B–D. Cytological analysis failed to detect cancer cells in 2 sequential PE samples of patient 28, a 42-year-old female, whose final diagnosis by tissue biopsy was malignant pleural mesothelioma (Figure 5B). As for patient 30, the PE sample was initially diagnosed as benign (cytologically negative) and was later determined as recurrent lung adenocarcinoma by thoracic lung needle aspiration (Figure 5C). The same negative cytologic results were found in PE of patient 93, who was ultimately diagnosed as histologically confirmed pleural metastasis of laryngeal cancer (Figure 5D). Notably, all 4 body fluid samples from these 3 patients were determined to be positive by the CUE model. Among the MBF samples with negative cytology, the CUE model yielded the highest positivity rate of 89.47% (34 out of 38) compared with other conventional tumor markers (≤50%), which revealed its potential as a clinical complementing measure of cytology in identifying malignancy (Figure 5E).

## Discussion

In this study, we characterize what we believe is a novel universal cancer–specific methylation marker, TAGMe. Along with the previously identified *PCDHGB7*, the 2 UCOM markers were first shown to be useful to detect cfDNA in body fluid samples. The CUE diagnostic model of the UCOM markers combined was subsequently developed and validated, which achieved promising performance in terms of diagnostic sensitivity, specificity, and cost effectiveness. This result is based on a prospective cohort that included 1,158 PE and ascites samples from patients with malignant and benign diseases. Further assessment of the extended cohort to detect malignancies in additional sample types such as CSF, PCE, and others (*n* = 163) demonstrated equal applicability.

Diagnosis of malignant tumors from body fluids is important to avoid more invasive examinations and enable prompt treatment. However, a major challenge is the constantly fluctuating sensitivity of routine cytology. For PE samples, 2 negative cytological results cannot exclude malignancies, as the diagnostic yield for malignancy of a first thoracentesis is 55%–60% (14). Studies of body-fluid-harvested materials (e.g., cancer antigens, cfDNA, microRNA, exosomes) have been reported to indicate early-stage tumors, monitor disease progression such as metastasis and recurrence, and detect chemoresistance (29–36). With the recent improvements in genomic technologies, identification of cfDNA in body fluid supernatant enables a more sensitive genetic detection, with minimal amounts of malignant cells (2, 37, 38). As indicated in this study, the clinical performance of the CUE model in discriminating between malignant and nonmalignant body fluids is superior to conventional tumor markers such as CEA, NSE, and CYFRA21-1 (AUC: 0.928 vs. 0.649, 0.708, and 0.746 in the test set).

In all cytology-negative MBFs from cancer patients, the diagnostic sensitivity of CUE reached 89.47% (34 out of 38). And the 4 samples that were tested negative by the CUE model were negative for other tumor markers as well. More importantly, in contrast to cytology’s sensitivity varying with the primary cancer, the performance of the CUE model was not significantly affected by cancer type (AUC: 0.885–0.952), with sensitivity in the range of 83.33%–97.73%. Moreover, performance evaluation on different sample types further showed the wide range of application scenarios (AUC: 0.909–1.000). Notably, diagnosis of malignant mesothelioma of the pleura has been a major clinical challenge, with extremely low clinical cytologic detection rates (28). Here, in PE and ascites samples from 20 malignant mesothelioma patients enrolled in this study, the overall sensitivity of the CUE model was 90%. Therefore, the CUE model was shown to improve the detectability of malignancy in body fluid specimens besides standard cytopathologic examination.

A limitation of our study is that the cohorts we used were enrolled in a single site, although the techniques of analysis and diagnostic criteria were generally applied across the country. In addition, as an observational study, not all patients with benign samples had histopathologic results, including some with positive CUE values, as they were diagnosed as nonmalignant based on available clinical tests and did not require a definitive biopsy. The underdiagnosis of tumor cells, if present, implies that the specificity of the CUE model may have been underestimated. Future follow-up studies are required to evaluate the fidelity of the CUE model’s clinical performance.

In conclusion, this is the largest prospective real-world study of UCOM in body fluid cfDNA. We developed and validated an MSRE-qPCR–based assay combining 2 UCOM markers, which are both cost effective and highly sensitive in detecting malignant cells. Moreover, the performance of the CUE model is consistent across different body fluid types and cancer types. Combined with its ability to detect cancer cells in cytologically negative MBFs, it suggests that it is a viable addition to current cytologic analyses.

## Methods

### Sex as a biological variable.

Our study included male and female participants, and similar findings are reported for both sexes.

### Study design and participants.

A total of 1,500 body fluid samples were collected from Zhongshan Hospital of Fudan University, and of these, 1,383 were eventually enrolled by exempting samples that met the exclusion criteria (Supplemental Figure 1). These included 62 retrospective PE samples from lung cancer patients and 1,321 prospective samples from various cancer and noncancer patients receiving diagnostic thoracic/abdominal paracentesis. All body fluid specimens had routine test results, such as cytological analysis, conventional cancer marker analysis, CT scan or/and histopathological examination, and were identified as malignant or nonmalignant ones based on guidelines of the Chinese Medical Association. After finishing the sample collection and methylation analysis in a double-blind manner, the clinical outcome was unblinded. Samples without a definitive pathological diagnosis were excluded from the study.

### Body fluid collection and cfDNA extraction.

As per guidelines (“Technical requirements for clinical body fluids analysis,” a Chinese standard for medical industry; WS/T 662-2020), 10 mL of PEs, ascites, PCEs, and BALF or 2 mL of CSF was taken from the sample’s remnants of protein and cytological analyses. After the cells and blood contaminations were removed by centrifugation, the cell-free supernatants were stored at –80°C for subsequent analysis. cfDNA extraction started with 1 mL of body fluid supernatant and was performed using the EP cfDNA Kit (Epiprobe Biotech) following the manufacturer’s instructions. In summary, 800 μL lysate (premixed with 1 μL carrier RNA [6 μg/μL] and 100 μL proteinase K [1 mg/mL]) were added to 1 mL body fluid supernatant and incubated at 60°C for 30 minutes. Then, 0.5 mL of isopropanol was added to the samples and incubated at room temperature for 5 minutes with 100 μL of carboxyl magnetic beads from the EP cfDNA Kit. Large fragments of DNA were bound to the beads and removed magnetically. Next, 200 μL of magnetic beads were added to the remaining supernatant, and then the cfDNA (>100 bp) was bound to the beads, washed twice, and eluted with 30 μL of ultrapure water. Finally, the cfDNA was quantified using the Qubit dsDNA HS Assay Kit (Thermo Fisher Scientific).

### cfDNA methylation detection.

Bisulfite pyrosequencing for the *PCDHGB7* genomic locus (chr5: 141,418,528–141,419,724, GRCh38/hg38) was conducted as previously described (39). The MSRE-qPCR was optimized based on the same study (39) to make it more suitable for cfDNA detection. In short, a calibration test targeting a conservative hypomethylated region with MSRE cutting sites was added for each PCR to rectify the disparity of digestion efficiency of MSRE in cfDNA samples. The subsequent triplex real-time quantification PCR was performed with the following program: initiation at 95°C for 10 minutes, and then 45 cycles of 94°C for 20 seconds and 60°C for 60 seconds. The UCOM marker identified in this study, located in chr3: 5,026,052–5,027,247 (GRCh38/hg38), is referred to as TAGMe. The TaqMan probe and primers were as follows: forward primer: 5′-TGGGGCCTGCACCCTAGA-3′, reverse primer: 5′-AGGAGACCAAGAGCATCCCG-3′; and probe: 5′-TTCCTGAGTGGGCCGTGC-3′.

### The Cancer Genome Atlas DNA methylation data analysis.

The Illumina 450K methylation array data from The Cancer Genome Atlas (TCGA) database was downloaded from the UCSC Xena browser (https://xenabrowser.net/ Accessed April 28, 2022.). The absolute methylation values were calculated from the β values of the 450K methylation array: methylation value (%) = (β value + 0.5) × 100. Six probes (cg02331883, cg02707176, cg04503600, cg13951490, cg13951490, and cg18297751) within the *PCDHGB7* region and 3 probes within the TAGMe (cg21545859, cg23516634, and cg03355909) region were selected. The final methylation value of each marker was calculated by the average of all selected probes. AUC of hypermethylated *PCDHGB7* and TAGMe and detailed information of all samples from TCGA project are listed in Supplemental Tables 2 and 3. The cancer types in which the target sites were not detected or the samples number was less than 3 in specific cancer or normal group were excluded from analysis.

### Statistics.

Statistical analyses and graphical visualization were conducted by using GraphPad Prism version 9.0 and Statistical Package for Social Sciences (SPSS) version 20.0 software (IBM). Nonparametric Mann-Whitney test was used to compare the differences between 2 groups. The ΔCt threshold of TAGMe and *PCDHGB7* was determined separately by constructing the ROC curve in the training set and Youden’s index was used to measure the diagnostic performance, which enables the selection of an optimal cutoff for each marker. Specifically, each cutoff was chosen separately when Youden’s index was maximized. The clinical performance of each marker was calculated using the corresponding cutoff point. And as for the conventional tumor markers, commonly used clinical thresholds by Zhongshan Hospital were adopted and are shown in Table 3. The 95%CI was calculated using the binomial distribution method. The differences between groups were assessed by a 2-tailed, nonparametric Mann-Whitney test or the Kruskal-Wallis test, and a *P* value of less than 0.05 was considered statistically significant. For all box-and-whisker plots, the upper and lower boundaries of the box, respectively, are the upper quartile (Q3) and lower quartile (Q1) of the data, the lines within the boxes represent the median of the data, the “+” within the boxes represents the mean, and the whiskers are the minimum to maximum values. The diagnostic model CUE was developed using binary logistic regression and data from the training set, and the CUE value of each sample was calculated as: CUE value = (1 + *e*^–[–^^ΔCtPCDHGB7^^×^^0.386^^–^^ΔCtTAGMe^^×^^0.974^^+^^5.822]^)^–1^.

### Study approval.

This study was approved by the ethics committees of Zhongshan Hospital, Fudan University (B2021-784R), and registered in the Chinese Clinical Trial Registry (ChiCTR2200060532). All study participants provided written informed consent.

### Data availability.

Data are available in the Supporting Data Values file in the supplemental material.

## Author contributions

WY, LT, YS, ZM, and SD designed the research study. YY, LW, YS, and LT recruited participants and collected the specimens and clinical information. ZM, SD, CW, and CQ conducted the experiments. ZM, SD, LT, and WY analyzed and interpreted the data. ZM wrote the manuscript. SD, WY, WL, LT, and CQ help revised the manuscript. WY, YS, WL, and LT provide administrative, technical, and material support. All authors approved the final manuscript version. The order of co–first authors was based on their relative contributions to the final version of the manuscript.

## Supplementary Material

Supplemental data

ICMJE disclosure forms

Supplemental table 3

Supporting data values

## Figures and Tables

**Table 1:**
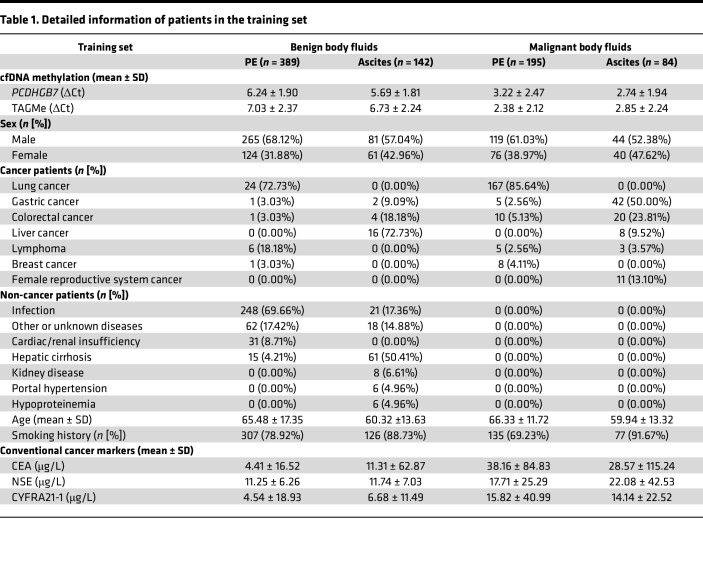
Detailed information of patients in the training set

**Table 2:**
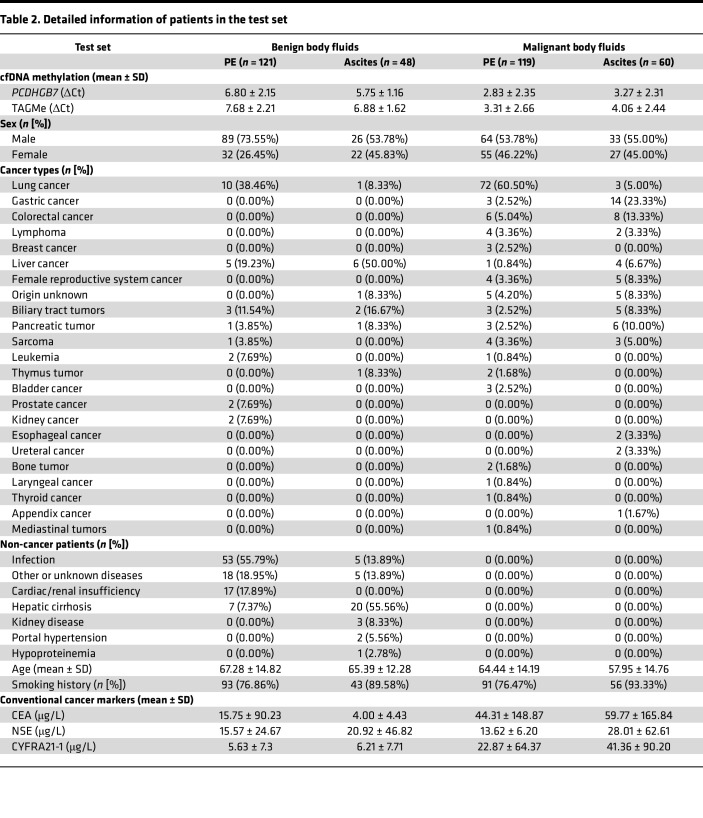
Detailed information of patients in the test set

**Table 3:**
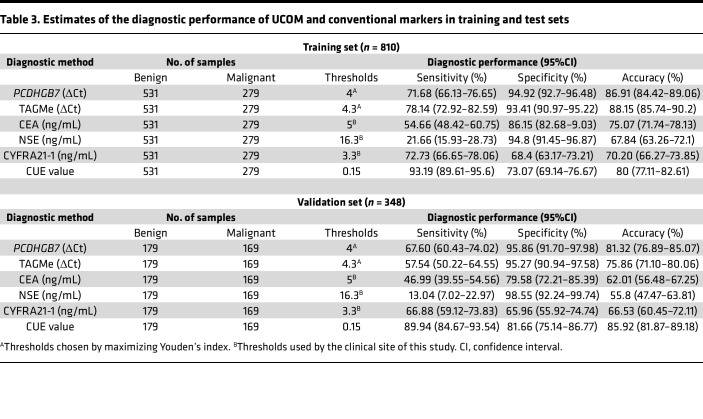
Estimates of the diagnostic performance of UCOM and conventional markers in training and test sets

## Version 1 04/08/2024

Electronic publication
