# Supplementary material for: Diagnosis of malignant body fluids via cancer-universal methylation in cell-free DNA
Source: JCI Insight. 2024 Apr 8;9(7):e175482. doi: 10.1172/jci.insight.175482 (PMC11128206; doi:10.1172/jci.insight.175482)
Supplement: Supplemental data [file jciinsight-9-175482-s021.pdf]

Supplemental Materials

Diagnosis of malignant body fluids via cancer-universal methylation in cell-free DNA

Zhanrui Mao [1#], Shihua Dong [2#], Yu Yan [3#], Chengyang Wang [2#], Wei Li [1], Lu Wang [3], Chengchen Qian [2], Yuanlin Song [3,4], Lin Tong [3,4*], Wenqiang Yu [1*]

[#]These authors contributed equally

Author Affiliations:

1. Institutes of Biomedical Sciences & Shanghai Public Health Clinical Center & Cancer Metastasis Institute & Department of General Surgery, Huashan Hospital, Shanghai Medical College, Fudan University, Shanghai, China.

2 Shanghai Epiprobe Biotechnology Co., Ltd, Shanghai, China.

3 Department of Pulmonary and Critical Care Medicine, Zhongshan Hospital, Fudan University, Shanghai, China.

4 Shanghai Respiratory Research Institute, Shanghai, China.

Corresponding Author:

Wenqiang Yu, PhD, Laboratory of RNA Epigenetics, Institutes of Biomedical Sciences & Shanghai Public Health Clinical Center & Department of General Surgery, Huashan Hospital & Cancer Metastasis Institute, Shanghai Medical College, Fudan University, Shanghai, 200032, China. (Tel: 15221889142, email: wenqiangyu@fudan.edu.cn)

Lin Tong, PhD, Department of Pulmonary and Critical Care Medicine, Zhongshan Hospital, Fudan University; Shanghai Respiratory Research Institute, Shanghai, 200032, China. (Tel: 18605373916, email: tong.lin@zs-hospital.sh.cn)

Supplementary information includes:

Supplementary methods

Figure S1. Diagram flow of patients.

Figure S2. The verification of UCOM markers in cell lines and tumor tissues.

Figure S3. cfDNA methylation of UCOM markers in training set.

Figure S4. cfDNA methylation of UCOM markers in extended set.

Table S1. Detailed information of 62 retrospective pleural effusion samples from lung cancer patients

Table S2. Area under the curve (AUC) of hypermethylated PCDHGB7 and TAGMe in different types of cancer in 450K methylation array data from TCGA database.

Table S3. Clinical information and methylation level of PCDHGB7 and TAGMe in samples from 450K methylation array data in TCGA database.

Table S4. Detailed information of patients in extended set

Table S5. Detailed information of patients with negative cytology and MBF that verified by definitive diagnosis.

Supplementary methods

Clinical samples

A total of 233 clinical samples across 8 types of cancer were collected from Zhongshan Hospital of Fudan University and Fudan University Shanghai Cancer Center, including breast cancer (cancer tissue: n = 11; paired para-cancer tissue: n = 11), cervical cancer (cancer tissue: n = 16; unpaired non-cancer tissue: n = 16), endometrial cancer (cancer tissue: n = 14; unpaired non-cancer tissue: n = 12); gastric cancer (cancer tissue: n = 5; unpaired para-cancer tissue: n = 5), lung cancer (cancer tissue: n = 27; unpaired para-cancer: n = 27), liver cancer (cancer tissue: n = 5; unpaired para-cancer tissue: n = 10), prostate cancer(cancer tissue: n = 15; unpaired non-cancer tissue: n = 15), urothelial cancer (cancer tissue: n = 11; unpaired non-cancer tissue: n = 12); white blood cells (WBCs) (from healthy individuals: n= 19), oral exfoliated epithelial cells (from healthy individuals: n= 2).

Cell lines

The cell lines including 5637, A549, A673, BIU87, CNE1, HCC-LM3, HeLa, HepG2, Huh-7, Jurkat, K562, MCFCA1a, MDA231-LM2-4175, MGC-803, MKN45, NCI-N87, SLKOV3, T24, THP-1 and U87MG were from our laboratory (Wenqiang Yu's lab at Fudan University). HepG2 was cultured in MEM (with NEAA) medium supplemented with 10% FBS and 1% Penicillin-Streptomycin; 5637, CNE1, Jurkat, THP-1 and MKN45 were cultured in RPMI-1640 medium with 10% FBS and 1% Penicillin-Streptomycin; while the others were cultured in DMEM with 10% FBS and 1% Penicillin-Streptomycin, at 37°C under 5% CO2.

DNA extraction

DNA extraction from frozen tissue and cell samples was conducted using EP Genomic DNA Kit (Epiprobe Biotech, K-21).

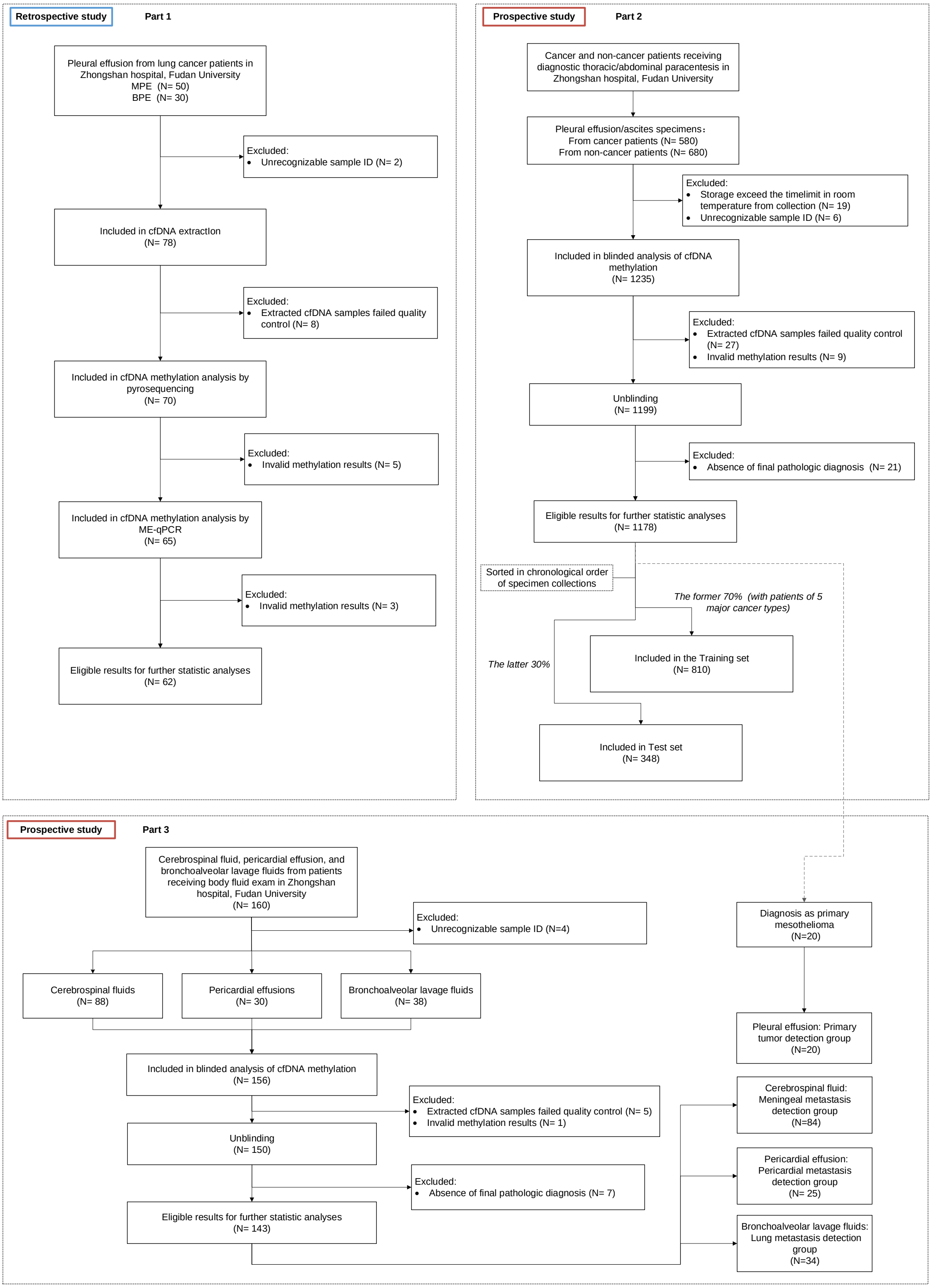

Figure S1. Diagram flow of patients.

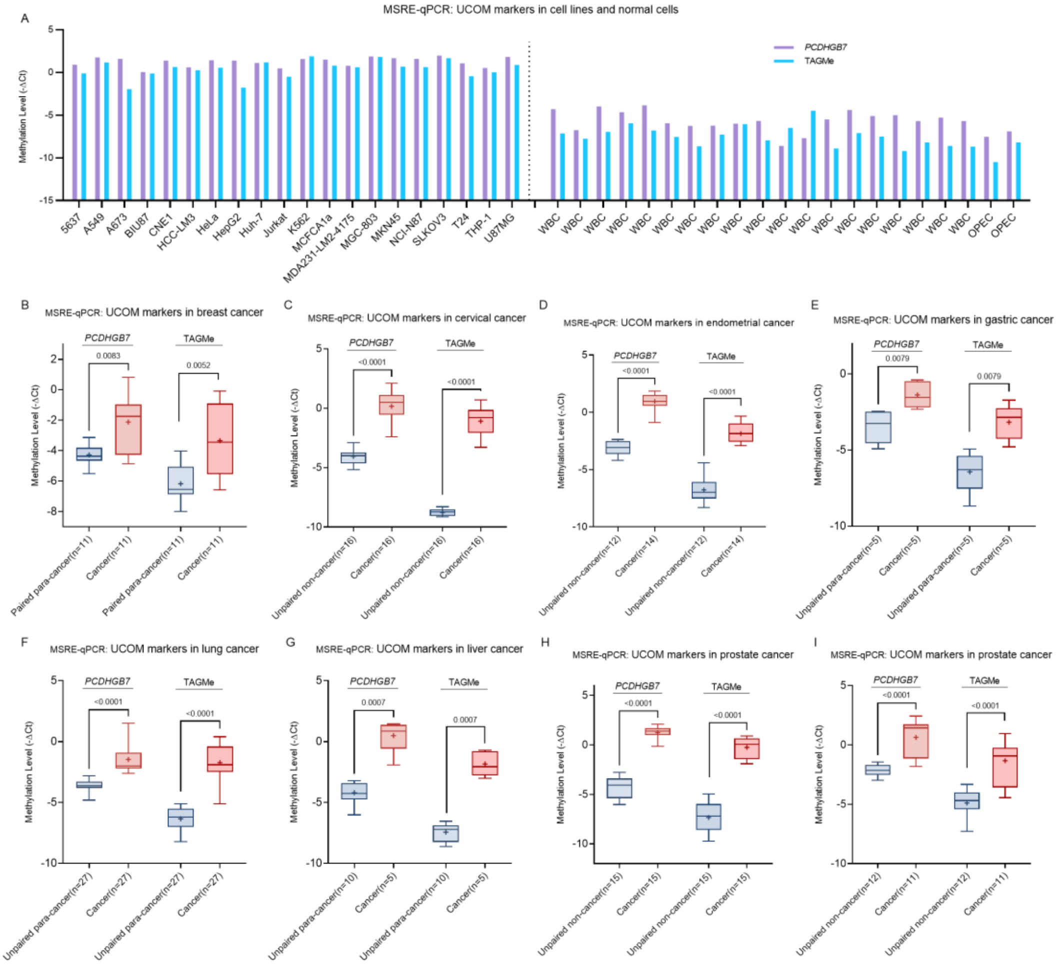

Figure S2. The verification of UCOM markers in cell lines and tumor tissues. The significant hypermethylation of UCOM markers was verified by MSRE-qPCR in multiple cancer cell lines (A). Methylation level of UCOM markers were significantly higher in tumor tissue of breast cancer (B), cervical cancer (C), endometrial cancer(D), gastric cancer(E), liver cancer (F), lung cancer (G), prostate cancer (H) and urothelial cancer (I) compared to normal controls. P values in (B-I) were calculated using a two-tailed nonparametric Mann-Whitney test as determined by GraphPad Prism 9.3.0.

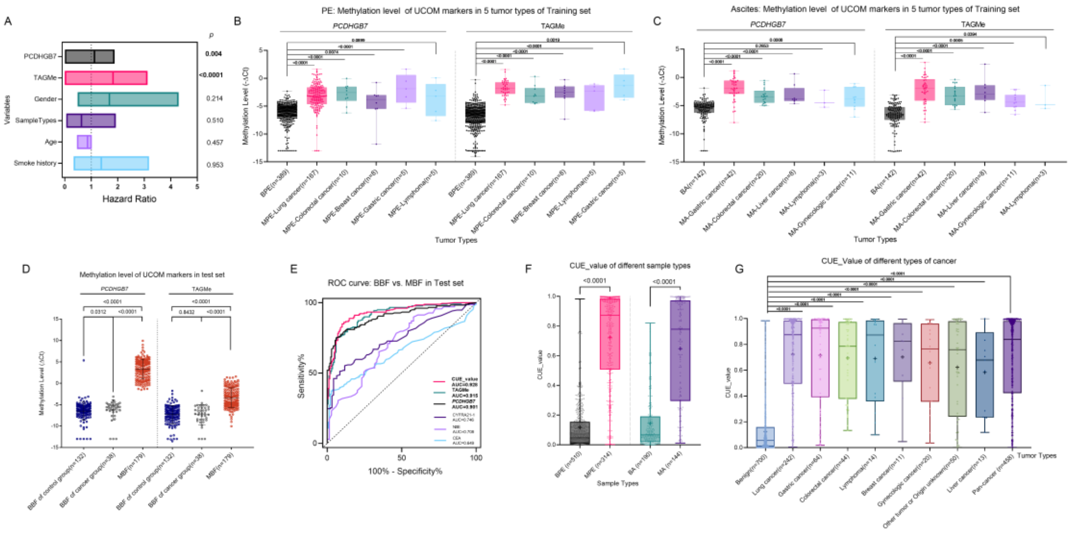

Figure S3. cfDNA methylation of UCOM markers in training set. Forest plot showing *PCDHGB7* and TAGMe are independent indicators for the detection of tumor cells in MBF (A). The methylation level of UCOM markers was significantly higher than that of BPE of different cancer types in training set (B). The methylation level of UCOM marker was significantly higher than that of BA of different cancer types in training set (C). Methylation levels of cfDNA UCOM marker also significantly associated with malignancy in test set (D). AUC of the CUE model and other markers were shown (E). Box plot showed the CUE value in training and test set by sample types (F) and cancer types (G). P values in (A) were determined by univariate Cox regression analysis with SPSS 20.0. P values in (B-D) and (G) were calculated using the Kruskal-Wallis test and in (F) were calculated by a two-tailed nonparametric Mann-Whitney test as determined by GraphPad Prism 9.3.0.

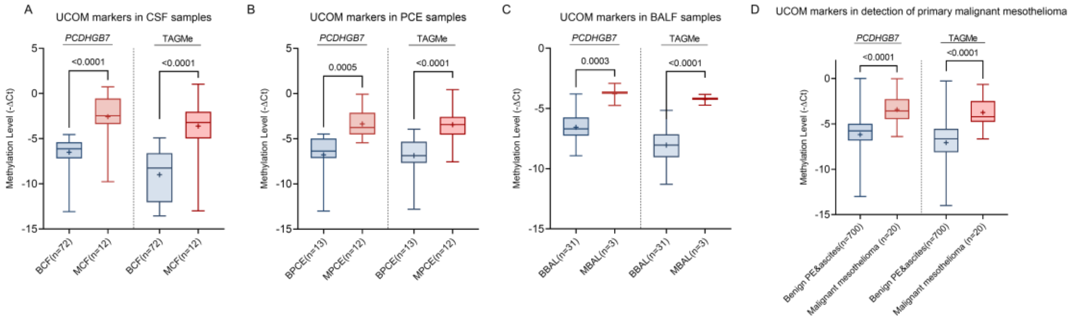

Figure S4. cfDNA methylation of UCOM markers in extended set. Methylation of two cfDNA UCOM markers were significantly associated with malignancies in CSF (A), PCE (B), BALF (C), and effusion samples of malignant mesothelioma patients (D). P values in (A-D) and (F-G) were calculated using a two-tailed nonparametric Mann-Whitney test as determined by GraphPad Prism 9.3.0.

Table S1. Detailed information of 62 retrospective pleural effusion samples from lung cancer patients

Sample_ID	Pyro-PCDHGB7(%)	MSRE-qPCR-PCDHGB7 (-ΔCt)	MSRE-qPCR-TAGMe (-ΔCt)	Gender	Age	Cytology	Classification
1	4.97	-5.85	-7.32	Female	79	Cancer cells	Non-malignant
2	6.04	-5.13	-7.72	Male	79	Cancer cells	Non-malignant
3	7.34	-4.82	-6.82	Female	50	Cancer cells	Non-malignant
4	7.90	-4.63	-6.71	Male	68	Cancer cells	Non-malignant
5	8.34	-3.99	-5.36	Male	64	Cancer cells	Non-malignant
6	8.56	-4.57	-5.80	Male	68	Cancer cells	Non-malignant
7	9.63	-4.40	-4.69	Male	76	Cancer cells	Non-malignant
8	10.04	-3.95	-5.63	Male	25	Abnormal cells	Non-malignant
9	10.51	-4.80	-6.49	Male	59	Cancer cells	Non-malignant
10	11.51	-4.08	-6.97	Female	64	Negative	Non-malignant
11	11.67	-3.93	-6.05	Female	61	Cancer cells	Non-malignant
12	12.30	-3.79	-4.26	Female	66	Cancer cells	Non-malignant
13	12.99	-4.15	-6.99	Male	57	Negative	Non-malignant
14	13.07	-4.07	-5.42	Female	64	Negative	Non-malignant
15	13.08	-4.67	-7.35	Male	56	Negative	Non-malignant
16	13.17	-4.50	-7.31	NA	NA	Negative	Non-malignant
17	13.24	-3.33	-5.19	Female	96	Negative	Non-malignant
18	13.32	-3.70	-5.96	Female	64	Negative	Non-malignant
19	13.52	-4.03	-6.18	Male	49	Abnormal cells	Non-malignant
20	14.89	-3.83	-4.44	Female	65	Negative	Non-malignant
21	15.19	-2.52	-4.72	Male	67	Negative	Non-malignant
22	15.81	-3.28	-3.32	Female	47	Negative	Malignant
23	16.57	-2.74	-3.27	NA	NA	Negative	Malignant
24	17.87	-1.83	-2.15	Female	70	Negative	Malignant
25	19.31	-0.96	-0.97	Female	47	Negative	Malignant
26	19.49	-2.73	-3.09	Male	78	Negative	Malignant
27	21.15	-0.70	-0.87	Female	66	Negative	Malignant
28	12.07	-4.04	-1.44	Male	44	Cancer cells	Malignant
29	14.32	-3.50	-4.50	Male	77	Cancer cells	Malignant
30	14.42	-4.11	-2.35	Male	57	Cancer cells	Malignant
31	15.02	-3.58	-1.15	Female	63	Cancer cells	Malignant
32	15.93	-3.45	-2.65	Male	72	Abnormal cells	Malignant
33	16.14	-3.91	-3.69	Male	60	Abnormal cells	Malignant
34	19.59	-2.97	-4.97	Male	34	Abnormal cells	Malignant
35	19.71	-2.20	-3.16	Female	60	Cancer cells	Malignant
36	19.75	-1.7	-3.60	Male	72	Abnormal cells	Malignant
37	20.04	-2.0	-4.23	Male	61	Abnormal cells	Malignant
38	20.06	-1.8	-3.80	Male	65	Cancer cells	Malignant
39	20.10	-1.2	-4.54	Female	NA	Cancer cells	Malignant

40	20.26	-1.0	-4.35	Female	45	Cancer cells	Malignant
41	20.85	-1.5	-3.16	Male	68	Cancer cells	Malignant
42	21.92	-1.23	-4.97	Male	64	Cancer cells	Malignant
43	22.17	-1.77	-4.17	Male	54	Cancer cells	Malignant
44	23.16	-1.40	-5.10	Female	67	Cancer cells	Malignant
45	24.02	-1.48	-4.06	Female	50	Cancer cells	Malignant
46	24.66	-1.04	-3.68	Female	42	Cancer cells	Malignant
47	25.11	-1.05	-3.48	Female	65	Cancer cells	Malignant
48	25.53	-1.5	-4.4	Male	53	Cancer cells	Malignant
49	25.91	-0.90	-4.31	Female	47	Cancer cells	Malignant
50	25.92	-0.22	-4.37	Male	58	Cancer cells	Malignant
51	29.03	-0.42	-0.32	Male	67	Cancer cells	Malignant
52	29.86	0.43	-3.66	Male	77	Cancer cells	Malignant
53	30.90	0.24	-0.62	Female	66	Abnormal cells	Malignant
54	31.25	0.19	-2.27	Female	55	Cancer cells	Malignant
55	36.06	0.30	-1.81	Male	65	Abnormal cells	Malignant
56	36.22	0.2	-2.3	Female	64	Cancer cells	Malignant
57	44.22	1.50	0.05	Female	69	Abnormal cells	Malignant
58	44.24	0.38	-2.11	Male	64	Cancer cells	Malignant
59	44.33	1.07	-2.56	Male	59	Cancer cells	Malignant
60	45.56	1.32	-0.74	Male	65	Cancer cells	Malignant
61	46.75	1.33	-2.37	Male	68	Cancer cells	Malignant
62	58.24	2.09	0.38	Male	65	Cancer cells	Malignant

Table S2. Area under the curve (AUC) of hypermethylated PCDHGB7 and TAGMe in different types of cancer in 450K methylation array data from TCGA database.

**Cancer Types**	**No. of samples**		**TAGMe**				**PCDHGB7**			
	Controls	Patients	AUC	95% confidence interval		*P* value	AUC	95% confidence interval		*P* value
**BLCA**	21	413	0.958	0.935	0.981	<.001	0.932	0.892	0.971	<.001
**BRCA**	98	774	0.982	0.974	0.99	<.001	0.939	0.922	0.955	<.001
**CESC**	18	309	1	1	1	0.0029	0.994	0.985	1	<.001
**CHOL**	9	36	0.883	0.78	0.986	<.001	0.83	0.711	0.949	0.0024
**COADREAD**	45	398	0.935	0.882	0.989	<.001	0.759	0.708	0.809	<.001
**ESCA**	16	186	0.86	0.73	0.99	<.001	0.851	0.702	0.999	<.001
**HNSC**	50	530	0.964	0.948	0.981	<.001	0.961	0.944	0.977	<.001
**KIRC**	160	320	0.864	0.832	0.897	<.001	0.888	0.856	0.92	<.001
**KIRP**	45	276	0.769	0.72	0.819	<.001	0.665	0.61	0.719	<.001
**LIHC**	50	379	0.806	0.767	0.844	<.001	0.916	0.886	0.945	<.001
**LUNG**	75	832	0.988	0.982	0.994	<.001	0.952	0.938	0.966	<.001
**PAAD**	10	185	0.931	0.865	0.998	<.001	0.925	0.855	0.995	<.001
**PRAD**	50	499	0.934	0.884	0.983	<.001	0.867	0.818	0.916	<.001
**UCEC**	46	432	0.954	0.936	0.972	<.001	0.977	0.965	0.989	<.001

BLCA, Bladder Cancer; BRCA, Breast Cancer; CESC, Cervical Cancer; CHOL, Bile Duct Cancer; COADREAD, Colon and Rectal Cancer; ESCA, Esophageal Cancer; GBM, Glioblastoma; HNSC, Head and Neck Cancer; KIRC, Kidney Clear Cell Carcinoma; KIRP, Kidney Papillary Cell Carcinoma; LIHC, Liver Cancer; LUNG, Lung Cancer; PAAD, Pancreatic Cancer; PRAD, Prostate Cancer; UCEC, Endometrioid Cancer.

Table S3. Clinical information and methylation level of PCDHGB7 and TAGMe in samples from 450K methylation array data in TCGA database.
Provided in an Excel (XLS) file.

Table S4. Detailed information of patients in extended set.

Sample _ID	PCDHGB7 (-ΔCt)	TAGMe (-ΔCt)	CUE_ value	Sample Types	Gender	Age	Classification of body fluids	Pathological diagnosis	Cancer types	Cytology
1	-5.43	-5.40	0.178	CSF	Male	60	Non-malignant	Viral Encephalitis	Non-cancer	Negative
2	-9.76	-2.13	0.496	CSF	Female	50	Malignant	Meningeal Metastases	Lung cancer	Negative
3	-2.60	-3.50	0.804	CSF	Male	38	Malignant	Meningeal Metastasis	Lung cancer	Abnormal cells
4	-3.83	-4.30	0.538	CSF	Female	72	Malignant	Meningeal metastasis	Lung cancer	Abnormal cells
5	-2.80	-2.70	0.892	CSF	Female	35	Malignant	Meningeal metastasis	Lung cancer	Abnormal cells
6	-2.32	-5.21	0.462	CSF	Male	65	Malignant	Meningeal Metastasis	Lung cancer	Cancer cells
7	-2.04	-13.00	0.000	CSF	Male	63	Malignant	Meningeal metastasis	Lung cancer	Cancer cells
8	-2.10	-3.44	0.840	CSF	Female	49	Malignant	Meningeal metastasis	Lung cancer	Cancer cells
9	0.26	0.49	0.998	CSF	Female	60	Malignant	Meningeal metastasis	Lung cancer	Cancer cells
10	-0.06	1.03	0.999	CSF	Male	54	Malignant	Meningeal Metastases	Colorectal cancer	Cancer cells
11	-2.70	-1.97	0.946	CSF	Female	72	Malignant	Meningeal Metastases	Lymphoma	Cancer cells
12	-3.59	-5.61	0.264	CSF	Female	51	Malignant	Primary CNS Lymphoma	Lymphoma	Cancer cells
13	0.74	-3.00	0.960	CSF	Female	34	Malignant	Meningeal Metastasis	Pontile tumor	Non-diagnostic
14	-5.86	-13.30	0.000	CSF	Female	59	Non-malignant	Non-meningeal metastases	Lung cancer	Negative
15	-4.73	-12.82	0.000	CSF	Male	45	Non-malignant	Non-Meningeal Metastases	Liver cancer	Negative
16	-8.06	-13.55	0.000	CSF	Male	67	Non-malignant	Non-meningeal metastases	Biliary tract tumors	Negative
17	-10.47	-5.83	0.020	CSF	Female	66	Non-malignant	Postresection	Glioblastoma	Negative
18	-5.46	-8.03	0.016	CSF	Male	67	Non-malignant	Non-Meningeal Metastases	Kidney cancer	Negative
19	-5.54	-7.38	0.029	CSF	Male	60	Non-malignant	Viral Encephalitis	Non-cancer	Negative
20	-6.78	-7.26	0.021	CSF	Male	21	Non-malignant	Viral Encephalitis	Non-cancer	Negative
21	-4.57	-13.06	0.000	CSF	Male	75	Non-malignant	Viral encephalitis	Non-cancer	Negative
22	-6.06	-8.72	0.007	CSF	Female	24	Non-malignant	Viral encephalitis	Non-cancer	Negative
23	-6.13	-8.89	0.005	CSF	Female	14	Non-malignant	Viral encephalitis	Non-cancer	Negative
24	-8.67	-13.28	0.000	CSF	Female	21	Non-malignant	Viral encephalitis	Non-cancer	Negative

25	-7.42	-6.59	0.030	CSF	Female	28	Non-malignant	Viral encephalitis	Non-cancer	Negative
26	-13.08	-6.12	0.006	CSF	Female	27	Non-malignant	Viral encephalitis	Non-cancer	Negative
27	-5.84	-6.37	0.067	CSF	Female	37	Non-malignant	Viral encephalitis	Non-cancer	Negative
28	-4.54	-6.83	0.070	CSF	Male	70	Non-malignant	Nervous system infection	Non-cancer	Negative
29	-6.12	-9.30	0.004	CSF	Male	70	Non-malignant	Nervous System Infections	Non-cancer	Negative
30	-7.48	-4.92	0.135	CSF	Male	79	Non-malignant	Nervous System Infections	Non-cancer	Negative
31	-6.77	-11.96	0.000	CSF	Male	42	Non-malignant	Nervous System Infections	Non-cancer	Negative
32	-5.38	-5.94	0.116	CSF	Male	34	Non-malignant	Nervous system infection	Non-cancer	Negative
33	-8.96	-6.02	0.029	CSF	Male	31	Non-malignant	Nervous System Infections	Non-cancer	Negative
34	-5.28	-6.64	0.064	CSF	Female	46	Non-malignant	Nervous System Infections	Non-cancer	Negative
35	-7.34	-8.11	0.007	CSF	Female	23	Non-malignant	Nervous System Infections	Non-cancer	Negative
36	-6.16	-8.39	0.009	CSF	Female	30	Non-malignant	Nervous System Infections	Non-cancer	Negative
37	-6.64	-6.55	0.042	CSF	Female	30	Non-malignant	Nervous System Infections	Non-cancer	Negative
38	-7.63	-7.42	0.013	CSF	Female	59	Non-malignant	Nervous System Infections	Non-cancer	Negative
39	-6.75	-6.64	0.037	CSF	Female	63	Non-malignant	Nervous System Infections	Non-cancer	Negative
40	-6.05	-7.27	0.027	CSF	Male	15	Non-malignant	Meningitis	Non-cancer	Negative
41	-7.72	-8.94	0.003	CSF	Female	54	Non-malignant	Meningitis	Non-cancer	Negative
42	-5.14	-9.91	0.003	CSF	Male	58	Non-malignant	Other benign diseases	Non-cancer	Negative
43	-7.25	-13.55	0.000	CSF	Male	44	Non-malignant	Other benign diseases	Non-cancer	Negative
44	-4.87	-12.21	0.000	CSF	Male	35	Non-malignant	Other benign diseases	Non-cancer	Negative
45	-12.00	-12.42	0.000	CSF	Male	54	Non-malignant	Other benign diseases	Non-cancer	Negative
46	-5.28	-12.36	0.000	CSF	Male	20	Non-malignant	Other benign diseases	Non-cancer	Negative
47	-6.06	-10.81	0.001	CSF	Male	67	Non-malignant	Other benign diseases	Non-cancer	Negative
48	-4.76	-7.14	0.049	CSF	Male	57	Non-malignant	Other benign diseases	Non-cancer	Negative

49	-5.20	-13.26	0.000	CSF	Male	62	Non-malignant	Other benign diseases	Non-cancer	Negative
50	-6.55	-12.98	0.000	CSF	Male	19	Non-malignant	Other benign diseases	Non-cancer	Negative
51	-4.94	-6.13	0.113	CSF	Male	48	Non-malignant	Other benign diseases	Non-cancer	Negative
52	-5.89	-12.38	0.000	CSF	Male	55	Non-malignant	Other benign diseases	Non-cancer	Negative
53	-6.97	-7.48	0.016	CSF	Male	66	Non-malignant	Other benign diseases	Non-cancer	Negative
54	-4.74	-5.18	0.258	CSF	Male	59	Non-malignant	Other benign diseases	Non-cancer	Negative
55	-5.39	-11.50	0.001	CSF	Male	57	Non-malignant	Other benign diseases	Non-cancer	Negative
56	-8.40	-11.08	0.000	CSF	Male	64	Non-malignant	Other benign diseases	Non-cancer	Negative
57	-6.39	-6.95	0.032	CSF	Male	70	Non-malignant	Other benign diseases	Non-cancer	Negative
58	-9.09	-9.32	0.001	CSF	Male	56	Non-malignant	Other benign diseases	Non-cancer	Negative
59	-5.40	-5.01	0.241	CSF	Female	66	Non-malignant	Other benign diseases	Non-cancer	Negative
60	-6.33	-13.04	0.000	CSF	Female	27	Non-malignant	Other benign diseases	Non-cancer	Negative
61	-5.22	-10.18	0.002	CSF	Female	67	Non-malignant	Other benign diseases	Non-cancer	Negative
62	-5.41	-11.75	0.000	CSF	Female	49	Non-malignant	Other benign diseases	Non-cancer	Negative
63	-7.60	-12.97	0.000	CSF	Female	74	Non-malignant	Other benign diseases	Non-cancer	Negative
64	-4.85	-10.42	0.002	CSF	Female	51	Non-malignant	Other benign diseases	Non-cancer	Negative
65	-5.20	-6.98	0.048	CSF	Female	49	Non-malignant	Other benign diseases	Non-cancer	Negative
66	-4.92	-6.95	0.055	CSF	Female	75	Non-malignant	Other benign diseases	Non-cancer	Negative
67	-6.59	-6.41	0.049	CSF	Female	80	Non-malignant	Other benign diseases	Non-cancer	Negative
68	-7.40	-6.81	0.025	CSF	Female	72	Non-malignant	Other benign diseases	Non-cancer	Negative
69	-6.63	-12.08	0.000	CSF	Female	62	Non-malignant	Other benign diseases	Non-cancer	Negative
70	-7.27	-5.35	0.100	CSF	Female	69	Non-malignant	Other benign diseases	Non-cancer	Negative

71	-5.79	-13.08	0.000	CSF	Female	74	Non-malignant	Other benign diseases	Non-cancer	Negative
72	-5.92	-12.65	0.000	CSF	Male	64	Non-malignant	Bacterial encephalitis	Non-cancer	Negative
73	-6.94	-13.28	0.000	CSF	Male	35	Non-malignant	Bacterial encephalitis	Non-cancer	Negative
74	-11.33	-5.30	0.024	CSF	Male	66	Non-malignant	Bacterial encephalitis	Non-cancer	Negative
75	-4.77	-8.81	0.010	CSF	Male	28	Non-malignant	Bacterial encephalitis	Non-cancer	Negative
76	-6.27	-10.89	0.001	CSF	Male	70	Non-malignant	Bacterial encephalitis	Non-cancer	Negative
77	-5.45	-8.95	0.007	CSF	Female	53	Non-malignant	Bacterial encephalitis	Non-cancer	Negative
78	-7.00	-11.54	0.000	CSF	Female	70	Non-malignant	Bacterial encephalitis	Non-cancer	Negative
79	-6.49	-6.10	0.067	CSF	Female	51	Non-malignant	Bacterial encephalitis	Non-cancer	Negative
80	-5.67	-4.93	0.237	CSF	Female	65	Non-malignant	Bacterial encephalitis	Non-cancer	Negative
81	-6.23	-6.68	0.043	CSF	Male	52	Non-malignant	Autoimmune encephalitis	Non-cancer	Negative
82	-5.68	-7.31	0.030	CSF	Male	70	Non-malignant	Autoimmune encephalitis	Non-cancer	Negative
83	-6.00	-6.39	0.062	CSF	Female	64	Non-malignant	Autoimmune encephalitis	Non-cancer	Negative
84	-6.67	-7.46	0.018	CSF	Female	82	Non-malignant	Autoimmune encephalitis	Non-cancer	Negative
85	-1.77	-2.42	0.942	PCE	Female	45	Malignant	Pericardial metastasis	Lung cancer	Cancer cells
86	-4.99	-4.54	0.372	PCE	Female	40	Non-malignant	Other benign diseases	Non-cancer	Negative
87	-5.44	-3.07	0.674	PCE	Female	57	Malignant	Pericardial metastasis	Lung cancer	Cancer cells
88	-4.19	-3.60	0.668	PCE	Female	56	Malignant	Pericardial metastasis	Lung cancer	Cancer cells
89	-4.48	-3.95	0.561	PCE	Female	62	Non-malignant	Cardiac insufficiency	Non-cancer	Negative
90	-2.08	0.43	0.996	PCE	Female	65	Malignant	Pericardial metastasis	Lung cancer	Abnormal cells
91	-4.66	-4.88	0.325	PCE	Male	53	Non-malignant	Cardiac insufficiency	Non-cancer	Negative
92	-5.27	-6.88	0.052	PCE	Male	78	Non-malignant	Cardiac insufficiency	Non-cancer	Negative

93	-4.96	-6.80	0.062	PCE	Male	56	Non-malignant	Nervous System Infections	Non-cancer	Negative
94	-3.58	-4.60	0.490	PCE	Male	69	Malignant	Pericardial metastasis	Lung cancer	Abnormal cells
95	-3.06	-4.39	0.591	PCE	Male	75	Malignant	Pericardial Metastasis	Lung cancer	Cancer cells
96	-3.95	-3.83	0.638	PCE	Female	61	Malignant	Pericardial Metastasis	Lung cancer	Negative
97	-7.47	-5.77	0.064	PCE	Female	70	Non-malignant	Other benign diseases	Non-cancer	Negative
98	-6.81	-7.02	0.025	PCE	Female	58	Non-malignant	Other benign diseases	Non-cancer	Negative
99	-6.26	-7.11	0.029	PCE	Male	40	Non-malignant	Nervous System Infections	Non-cancer	Negative
100	-4.63	-3.23	0.709	PCE	Male	58	Malignant	Pericardial metastasis	Lung cancer	Cancer cells
101	-13.00	-12.80	0.000	PCE	Male	30	Non-malignant	Other benign diseases	Non-cancer	Negative
102	-6.37	-7.28	0.023	PCE	Female	51	Non-malignant	Other benign diseases	Non-cancer	Negative
103	-5.30	-5.00	0.250	PCE	Male	43	Malignant	Pericardial Metastasis	Mediastinal malignant tumor	Cancer cells
104	-0.08	-0.83	0.993	PCE	Male	55	Malignant	Pericardial metastasis	Lung cancer	Cancer cells
105	-4.03	-3.28	0.746	PCE	Female	49	Malignant	Pericardial Metastasis	Lung cancer	Negative
106	-6.67	-8.07	0.010	PCE	Male	50	Non-malignant	Other benign diseases	Non-cancer	Negative
107	-10.81	-5.94	0.016	PCE	Female	57	Non-malignant	Nervous System Infections	Non-cancer	Negative
108	-2.29	-7.56	0.082	PCE	Female	64	Malignant	Pericardial Metastasis	Lung cancer	Cancer cells
109	-6.48	-8.17	0.010	PCE	Male	63	Non-malignant	Other benign diseases	Non-cancer	Negative
110	-7.70	-8.04	0.007	BALF	Male	73	Non-malignant	Interstitial lung disease	Non-cancer	Negative
111	-5.43	-7.53	0.026	BALF	Female	67	Non-malignant	Interstitial lung disease	Non-cancer	Negative
112	-7.96	-9.29	0.002	BALF	Male	68	Non-malignant	Interstitial lung disease	Non-cancer	Negative
113	-2.91	-4.19	0.649	BALF	Male	56	Malignant	Lung Lymphoma	Lymphoma	Non-diagnostic
114	-6.54	-6.99	0.029	BALF	Male	55	Non-malignant	Immunotherapy-associated pneumonia	Lung cancer	Negative

115	-5.37	-7.15	0.039	BALF	Male	58	Non-malignant	Postresection	Pancreatic cancer	Negative
116	-5.82	-9.34	0.004	BALF	Female	65	Non-malignant	Interstitial lung disease	Non-cancer	Negative
117	-6.99	-11.29	0.000	BALF	Male	76	Non-malignant	Interstitial lung disease	Non-cancer	Negative
118	-5.75	-9.26	0.004	BALF	Male	53	Non-malignant	Interstitial lung disease	Non-cancer	Negative
119	-6.69	-7.73	0.014	BALF	Female	59	Non-malignant	Interstitial lung disease	Non-cancer	Negative
120	-7.11	-8.88	0.004	BALF	Male	58	Non-malignant	Other benign diseases	Non-cancer	Negative
121	-6.54	-6.84	0.034	BALF	Female	51	Non-malignant	Interstitial lung disease	Non-cancer	Negative
122	-5.70	-6.30	0.075	BALF	Female	55	Non-malignant	Interstitial lung disease	Non-cancer	Negative
123	-6.09	-9.05	0.005	BALF	Female	79	Non-malignant	Pleural Metastases	Lung cancer	Negative
124	-6.50	-7.59	0.017	BALF	Male	33	Non-malignant	Interstitial lung disease	Non-cancer	Negative
125	-6.83	-7.61	0.014	BALF	Female	55	Non-malignant	Interstitial lung disease	Non-cancer	Negative
126	-6.64	-7.17	0.024	BALF	Male	71	Non-malignant	Immunotherapy-associated pneumonia	Lung cancer	Negative
127	-8.83	-8.49	0.003	BALF	Female	57	Non-malignant	Interstitial lung disease	Non-cancer	Negative
128	-4.04	-8.04	0.027	BALF	Male	58	Non-malignant	Interstitial lung disease	Non-cancer	Negative
129	-6.90	-8.75	0.005	BALF	Male	72	Non-malignant	Other benign diseases	Non-cancer	Negative
130	-8.92	-9.39	0.001	BALF	Male	58	Non-malignant	Interstitial lung disease	Non-cancer	Negative
131	-7.64	-8.64	0.004	BALF	Male	48	Non-malignant	Interstitial lung disease	Non-cancer	Negative
132	-7.29	-8.92	0.003	BALF	Male	51	Non-malignant	Interstitial lung disease	Non-cancer	Negative
133	-6.84	-5.94	0.069	BALF	Male	75	Non-malignant	Interstitial lung disease	Non-cancer	Negative
134	-5.72	-7.60	0.022	BALF	Male	77	Non-malignant	Acute Respiratory Distress Syndrome (ARDS)	Esophageal cancer	Negative
135	-3.79	-5.16	0.340	BALF	Female	68	Non-malignant	Interstitial Lung Disease	Lymphoma	Negative

136	-6.05	-5.95	0.090	BALF	Male	26	Non-malignant	Acute Respiratory Distress Syndrome (ARDS)	Lymphoma	Negative
137	-4.74	-4.73	0.350	BALF	Female	50	Malignant	Lung Metastases	Breast cancer	Cancer cells
138	-7.25	-9.14	0.003	BALF	Male	56	Non-malignant	Lung Infection	Lung cancer	Negative
139	-7.02	-8.72	0.005	BALF	Female	63	Non-malignant	Immunotherapy-associated pneumonia	Colorectal cancer	Negative
140	-4.97	-6.22	0.103	BALF	Male	69	Non-malignant	Interstitial Lung Disease	Lymphoma	Negative
141	-3.68	-3.83	0.662	BALF	Male	81	Malignant	Lung metastases	Lung cancer	Cancer cells
142	-7.39	-9.25	0.002	BALF	Female	58	Non-malignant	Other benign diseases	Non-cancer	Negative
143	-7.19	-9.02	0.003	BALF	Female	39	Non-malignant	Other benign diseases	Non-cancer	Negative
144	-5.36	-4.64	0.318	PE	Male	66	Malignant	Primary pleural tumor	Pleural mesothelioma	Negative
145	-1.66	-2.35	0.948	PE	Male	27	Malignant	Primary peritoneal tumor	Peritoneal mesothelioma	Abnormal cells
146	-3.82	-3.55	0.708	PE	Female	42	Malignant	Primary pleural tumor	Pleural mesothelioma	Negative
147	-4.25	-4.24	0.512	PE	Male	82	Malignant	Primary pleural tumor	Pleural mesothelioma	Cancer cells
148	-1.88	-1.75	0.967	PE	Male	56	Malignant	Primary pleural tumor	Pleural mesothelioma	Abnormal cells
149	-1.11	-1.35	0.983	PE	Male	83	Malignant	Primary pleural tumor	Pleural mesothelioma	Cancer cells
150	-4.21	-3.88	0.604	PE	Female	74	Malignant	Primary pleural tumor	Pleural mesothelioma	Abnormal cells
151	-4.99	-2.88	0.749	Ascite	Female	63	Malignant	Primary peritoneal tumor	Peritoneal mesothelioma	Negative
152	-3.59	-0.65	0.978	Ascite	Male	67	Malignant	Primary peritoneal tumor	Peritoneal mesothelioma	Cancer cells
153	-2.68	-4.18	0.672	PE	Male	57	Malignant	Primary pleural tumor	Pleural mesothelioma	Cancer cells
154	-3.20	-4.72	0.496	PE	Female	42	Malignant	Primary pleural tumor	Pleural mesothelioma	Negative
155	-2.13	-2.90	0.898	PE	Male	55	Malignant	Primary pleural tumor	Pleural mesothelioma	Abnormal cells
156	-4.55	-5.01	0.306	Ascite	Male	85	Malignant	Primary peritoneal tumor	Peritoneal mesothelioma	Negative
157	-3.55	-4.45	0.530	PE	Male	52	Malignant	Primary peritoneal tumor	Peritoneal mesothelioma	Cancer cells

158	-6.40	-6.65	0.042	PE	Female	42	Malignant	Primary pleural tumor	Pleural mesothelioma	Negative
159	-0.03	-1.28	0.990	Ascite	Female	80	Malignant	Primary peritoneal tumor	Peritoneal mesothelioma	Cancer cells
160	-3.09	-4.57	0.544	PE	Female	71	Malignant	Primary pleural tumor	Pleural mesothelioma	Cancer cells
161	-3.93	-5.57	0.246	PE	Female	68	Malignant	Primary pleural tumor	Pleural mesothelioma	Cancer cells
162	-3.54	-5.39	0.312	Ascite	Male	42	Malignant	Primary peritoneal tumor	Peritoneal mesothelioma	Cancer cells
163	-4.60	-4.80	0.347	PE	Male	81	Malignant	Primary pleural tumor	Pleural mesothelioma	Abnormal cells

Table S5. Detailed information of patients with negative cytology and MBF that verified by definitive diagnosis.

ID	PCDHGB7 (-ΔCt**)**	TAGMe (-ΔCt**)**	CUE_ Value	Sample Type	Gender	Age	Classification of body fluids	Diagnosis	CEA	NSE	CYFR A21-1
Patient_ 13	-4.62	-6.22	0.12	Ascite	Male	49.0	Malignant	Peritoneal metastasis of liver cancer	1.7	11.7	2.4
Patient_ 136	-9.76	-2.13	0.50	CSF	Female	50.0	Malignant	Meningeal metastases of lung adenocarcinoma	1.4	0.0	N
Patient_ 142	-3.22	-5.73	0.27	PE	Male	68.0	Malignant	Pleural metastasis of mucosa-associated lymphoid tissue (MALT) lymphoma	3.1	13.9	3.3
Patient_ 171	-4.55	-5.01	0.31	Ascite	Male	85.0	Malignant	Malignant peritoneal mesothelioma	2.8	0.0	N
Patient_ 181	-4.11	-3.45	0.70	PE	Male	56.0	Malignant	Pleural metastasis of lung cancer	N	34.0	N
Patient_ 2	-5.36	-4.64	0.32	PE	Male	66.0	Malignant	Malignant pleural mesothelioma	6.1	N	9.4
Patient_ 23	-7.58	-2.27	0.66	PE	Male	71.0	Malignant	Waldenstrom's macroglobuline mia	N	N	N
Patient_ 232	3.62	3.05	1.00	PE	Female	76.0	Malignant	Pleural metastasis of pancreatic cancer	3.1	N	53.2
Patient_ 242	-0.07	0.57	1.00	PE	Female	46.0	Malignant	Pleural metastasis of B-cell lymphoma	0.8	N	1.5
Patient_ 248-1	-4.03	-3.61	0.68	Ascite	Male	70.0	Malignant	Peritoneal metastasis of liver cancer	5.7	11.4	3.3
Patient_ 248-2	-4.21	-2.67	0.83	Ascite	Male	70.0	Malignant	Peritoneal metastasis of liver cancer	5.7	11.4	3.3
Patient_ 248-3	-3.91	-1.29	0.96	Ascite	Male	70.0	Malignant	Peritoneal metastasis of liver cancer	5.7	11.4	3.3
Patient_ 255	-6.61	-2.86	0.62	PE	Female	82.0	Malignant	Pleural metastasis of lung adenocarcinoma	7.8	14.5	2.2
Patient_ 279	-6.40	-6.65	0.04	PE	Female	42.0	Malignant	Malignant pleural mesothelioma	0.9	9.2	1.5
Patient_ 28-1	-3.82	-3.55	0.71	PE	Female	42.0	Malignant	Malignant pleural mesothelioma	N	N	N
Patient_ 28-2	-3.20	-4.72	0.50	PE	Female	42.0	Malignant	Malignant pleural mesothelioma	2.3	9.3	1.5
Patient_ 297	-6.38	-2.24	0.76	Ascite	Female	33.0	Malignant	Peritoneal and lungs metastasis of pelvic sarcomas	0.6	N	1.5
Patient_ 30	-4.42	-4.07	0.54	PE	Female	76.0	Malignant	lung adenocarcinoma	37.0	N	45.0
Patient_ 318	-6.45	-6.22	0.06	Ascite	Female	79.0	Malignant	Peritoneal metastasis of sarcoma	1.5	14.1	2.0

Patient_362	-6.21	-2.91	0.64	PE	Female	65.0	Malignant	Pleural metastasis of colorectal cancer	2.5	N	22.3
Patient_370	-4.99	-2.88	0.75	Ascite	Female	63.0	Malignant	Malignant peritoneal mesothelioma	1.8	12.5	2.2
Patient_39	-0.60	-2.18	0.97	PE	Female	84.0	Malignant	Pleural metastasis of lung cancer	2.8	N	13.4
Patient_47	-5.61	-5.85	0.11	Ascite	Female	70.0	Malignant	Peritoneal metastasis of ovarian serous carcinoma	N	N	N
Patient_486	-4.76	-4.81	0.33	PE	Male	64.0	Malignant	Pleural metastasis of lung cancer	5.2	N	8.5
Patient_508	-3.95	-3.83	0.64	PCE	Female	61.0	Malignant	Pericardial metastasis of lung adenocarcinoma	157.0	N	7.2
Patient_520	-4.63	-4.48	0.42	PE	Male	81.0	Malignant	Pleural metastasis of lung squamous cell carcinoma	9.9	N	91.4
Patient_524	-5.09	-3.48	0.62	PE	Female	58.0	Malignant	Pleural metastasis of lung cancer	2.8	13.0	2.1
Patient_539	-3.23	-0.96	0.97	Ascite	Male	64.0	Malignant	Peritoneal metastasis of sigmoid colon cancer	>1000	67.8	92.7
Patient_54	-5.44	-2.21	0.83	PE	Female	43.0	Malignant	Pleural metastasis of breast cancer	16.6	N	96.0
Patient_57	3.44	5.22	1.00	PE	Female	30.0	Malignant	Pleural metastasis of bone cancer	0.8	15.8	2.5
Patient_73	4.27	3.02	1.00	PE	Female	52.0	Malignant	Pleural metastasis of pelvic malignancies	1.4	10.3	1.8
Patient_780	-4.03	-3.28	0.75	PCE	Female	49.0	Malignant	Pericardial, brain and bone metastasis of lung adenocarcinoma	15.3	25.4	23.6
Patient_792	3.33	4.44	1.00	PE	Male	68.0	Malignant	pericardial and pleural metastases of bladder cancer	4.6	N	1.2
Patient_817	-3.00	-1.38	0.96	PE	Female	68.0	Malignant	Pleural metastasis of breast cancer	1.1	N	1.3
Patient_829	-4.26	-5.44	0.25	Ascite	Male	59.0	Malignant	Peritoneal metastasis of liver cancer	1.8	N	N
Patient_833	0.05	0.41	1.00	PE	Male	66.0	Malignant	Pleural and lymph node metastasis of non-small cell lung cancer	41.4	24.5	67.9
Patient_91	-2.25	-2.84	0.90	PE	Male	56	Malignant	Pleural metastasis of lung cancer	5.7	15.8	16.1
Patient_93	2.40	4.70	1.00	PE	Male	62	Malignant	Pleural metastasis of laryngeal cancer	17.8	15.9	43.0
